# Particulate matter exposure during pregnancy and birth outcomes: exposure windows of susceptibility and socioeconomic inequalities

**DOI:** 10.1007/s10654-025-01274-1

**Published:** 2025-07-24

**Authors:** Mònica Guxens, Núria Botella, Massimo Stafoggia, Marcelle Canto, Sami Petricola, Antònia Valentín, Aitana Lertxundi, Ana Fernández-Somoano, Carmen Freire, Anna García-Altés, Elia Diez, Marc Marí-Dell’Olmo, Carmen Iñiguez, María José López, Rebeca Ramis, Anne-Claire Binter

**Affiliations:** 1 ISGlobal, Barcelona, Spain; 2 Universitat Pompeu Fabra, Barcelona, Spain; 3 Spanish Consortium for Research on Epidemiology and Public Health (CIBERESP), Instituto de Salud Carlos III, Madrid, Spain; 4 Department of Child and Adolescent Psychiatry/Psychology, Erasmus MC, University Medical Centre, Rotterdam, The Netherlands; 5 ICREA, Barcelona, Spain; 6 Department of Epidemiology, Lazio Region Health Service ASL ROMA 1, Rome, Italy; 7 Doctoral Program in Biomedical Sciences and Public Health, International Doctorate Program, National University of Distance Education (UNED), Madrid, Spain; 8 Department of Preventive Medicine and Quality Management, Hospital General Universitario Gregorio Marañon, Madrid, Spain; 9 Department of Preventive Medicine and Public Health, University of the Basque Country (UPV/EHU), Leioa, Bizkaia Spain; 10 Group of Public Health and Environmental Epidemiology, Biogipuzkoa Health Research Institute, Donostia-San Sebastián, Spain; 11 Department of Medicine, University Institute of Oncology of the Principality of Asturias (IUOPA), University of Oviedo, Asturias, Spain; 12 Department of Legal Medicine, Toxicology, and Physical Anthropology, University of Granada, Granada, Spain; 13 Instituto de Investigación Biosanitaria de Granada (ibs.granada), Granada, Spain; 14 Agency for Heath Quality and Assessment of Catalonia (AQuAS), Barcelona, Spain; 15 Public Health Agency of Barcelona, Barcelona, Spain; 16 Institut de Recerca Sant Pau (IR SANT PAU), Barcelona, Spain; 17 Department of Statistics and Operational Research, Universitat de València, Valencia, Spain; 18 Cancer and Environmental Epidemiology Unit, Chronic Diseases Department, National Centre for Epidemiology, Carlos III Institute of Health, Madrid, Spain

**Keywords:** Air pollution, Maternal exposure, Birth weight, Preterm birth, Small for gestational age, Socioeconomic disparities in health

## Abstract

We aimed (i) to assess the relationship of pregnancy-average particulate matter (PM) exposure with birthweight, birthweight at term, low birthweight at term, small for gestational age, and preterm birth, (ii) to identify critical windows of susceptibility to PM exposure across pregnancy on birthweight and small for gestational age, and (iii) to assess the presence of socioeconomic inequalities on these associations. We established a population-based, nationwide cohort using the Spanish birth registry between 2004 and 2016 (*N* = 3,678,445). We estimated daily PM_10_ and PM_2.5_ concentrations for the entire pregnancy at the maternal residential address at child’s delivery using spatiotemporal land use random-forest models. Linear, logistic, and distributed lag linear models were used for the different analysis. All models were stratified by maternal educational level and area-level deprivation index. Mean PM_10_ and PM_2.5_ concentrations during pregnancy were 25.1 and 12.7 µg/m^3^, respectively. Higher pregnancy-average PM_10_ concentrations were associated with lower birthweight and increased odds of preterm birth (-7.1 g [95%CI -8.5; -5.7] and OR 1.04 [95%CI 1.02; 1.05], respectively, per 10 µg/m^3^ increase in PM_10_). Similar results were found for PM_2.5_, in particular for levels above 10 µg/m^3^. These associations were stronger in infants born to mothers with lower education, particularly when combined with residence in more deprived areas. We observed some windows of susceptibility to PM_10_ for birthweight, mainly in the third trimester of pregnancy, with a similar pattern across socioeconomic levels. We did not observe windows of susceptibility to PM_2.5_. Structural policies to reduce exposure to current PM levels in pregnant women and socioeconomic inequalities are needed.

**Supplementary Information:**

The online version contains supplementary material available at 10.1007/s10654-025-01274-1.

## Introduction

Over recent decades, numerous regulatory policies have been implemented across Europe to reduce air pollution levels [1]. Despite these efforts, air pollution remains the leading environmental health challenge, constituting the primary environmental contributor to the global burden of disease and one of the most significant preventable causes of morbidity and mortality [2, 3]. Pregnant women and their fetuses represent particularly vulnerable populations. During pregnancy, rapid fetal development renders the fetus highly susceptible to environmental insults, including air pollution. Fine particulate matter (PM) can deposit in the maternal respiratory tract, with soluble components potentially translocating into the bloodstream, thereby inducing systemic inflammation [4, 5]. This inflammatory response may adversely affect fetal development through various biological mechanisms [4, 5].

An extensive body of literature has shown that prenatal air pollution exposure, in particular PM with aerodynamic diameter < 2.5 μm (PM_2.5_), is associated with adverse birth outcomes including lower birthweight, preterm birth, and small for gestational age [4–14]. However, fewer studies have attempted to identify critical windows of susceptibility to PM exposure across pregnancy in relation to birthweight and small for gestational age using fine temporal resolution air pollution estimates instead of exploring a priori defined periods such as trimesters of pregnancy [15–21]. Revealing such susceptible periods is essential for informing both clinical practice and public health policy aimed at safeguarding fetal growth and development. With regard to lower birthweight, most studies have identified the second and/or third trimester as periods of increased susceptibility [15–19]. In contrast, findings related to small for gestational age have been inconsistent [18, 20, 21].

In addition, socioeconomic status is a well-established determinant of adverse birth outcomes [22] and has also been associated with differential exposure to air pollution [23]. Beyond the confounding role of socioeconomic status, it is crucial to investigate whether it modifies the relationship between air pollution exposure during pregnancy and birth outcomes. Such an understanding is essential for designing targeted and equitable interventions. However, it has been insufficiently investigated. A limited number of studies suggest that infants born to African-American or Black mothers and those born to mothers with lower educational attainment may be disproportionately affected [24, 25]. However, mixed results have been shown on the identification of windows of susceptibility to PM exposure by socioeconomic factors [18, 26, 27].

Therefore, we aim to assess the relationship between pregnancy-average PM exposure and birthweight, birthweight at term, low birthweight at term, small for gestational age, and preterm birth. In addition, we aim to identify critical windows of susceptibility to PM exposure across pregnancy on birthweight and small for gestational age. Finally, we aim to assess the presence of socioeconomic inequalities on the association between prenatal exposure to PM and birth outcomes.

## Methods

### Study population

**Fig. 1:**
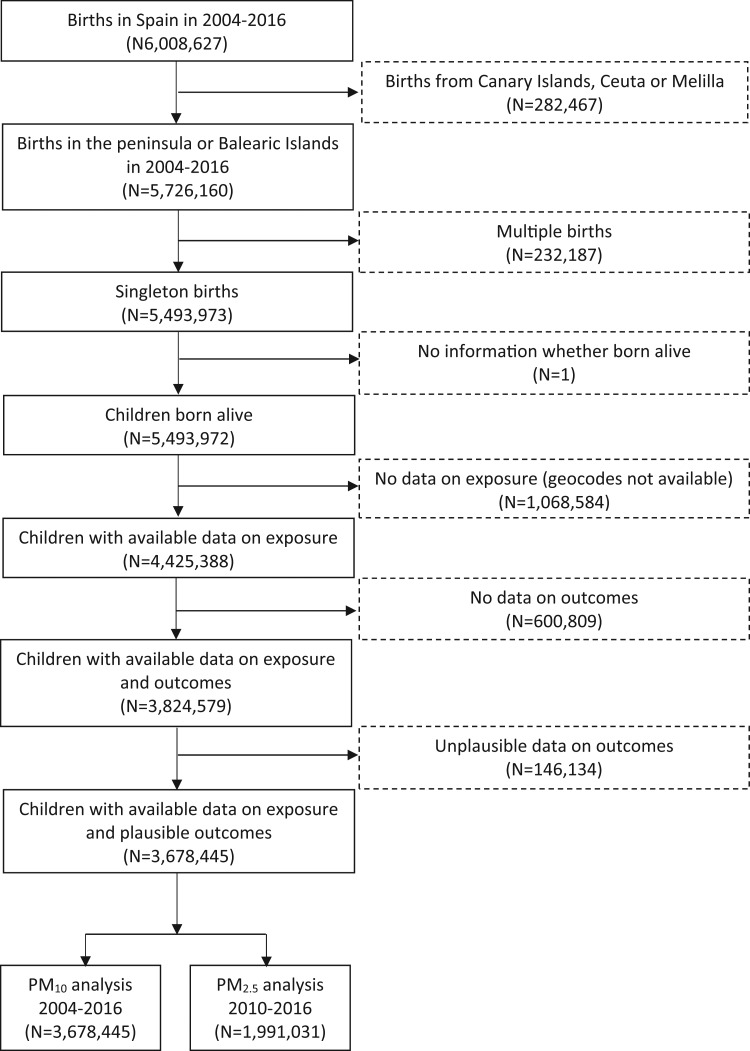
Flowchart of study participants

We established a nationwide, population-based cohort using the Spanish Birth Registry Statistics database of the National Statistics Institute. The initial population comprised singleton live births recorded across Spain (excluding Canary Islands, Ceuta, and Melilla) between January 2004 and December 2016 (*N* = 5,493,972) (Fig. 1). We excluded births with missing information on air pollution exposure (due to unavailable maternal address at delivery), birthweight, gestational age, and implausible values for birthweight or gestational age. This resulted in a final sample of 3,678,445 birth records (67% of the initial population). We only had data on PM_2.5_ between 2009 and 2016, thus the final sample involving PM_2.5_ included 1,991,031 births from January 2010 to December 2016.

### Air pollution concentration measures

We developed spatiotemporal land use random-forest models to estimate daily concentrations of PM_10_ and PM_2.5_ across peninsular Spain and the Balearic Islands following a methodology previously applied in Italy and Sweden [28, 29] (Methods S1). Briefly, these models estimated daily concentrations of PM_10_ between 2003 and 2016 and PM_2.5_ between 2009 and 2016 over 1  x 1 km grid cells using spatiotemporal and spatial predictors. Daily PM concentrations were obtained from the national air quality database. Spatiotemporal predictors included daily remote sensing data on aerosol optical depth, meteorological data, normalized difference vegetation index, and Saharan dust advections. Spatial predictors included light at night, impervious surface area, elevation, road, administrative boundaries, land cover data, slope, population, climate classification, and phytoclimatic types.

Maternal residential addresses at delivery were geocoded by the National Statistics Institute and anonymized by randomly allocating coordinates within a 30 m buffer. Daily PM_10_ and PM_2.5_ concentrations were estimated at these geocoded locations for each day of gestation (i.e., from conception until birth) using small-scale temporal and spatial predictors. We assumed that women did not change their residence during pregnancy. Daily PM_10_ and PM_2.5_ concentrations were aggregated to calculate pregnancy-average and weekly-average exposures.

### Birth outcomes

Information on birthweight (in grams) and gestational age (in weeks) were obtained from the birth registry. We calculated preterm birth, birthweight at term, low birthweight at term, and small for gestational age. Preterm birth was defined as having a gestational age at birth lower than 37 weeks. Birthweight at term was defined as birthweight restricted to those children born from week 37 onwards [5]. Low birthweight at term was defined as having a birthweight below 2,500 g for those children born from week 37 onwards [5]. Small for gestational age was defined as having a birthweight below the 10th percentile considering its gestational age and sex [5].

### Potential confounding variables

The birth registry provided data on maternal and paternal age (years), educational level (low, medium, high based on primary or lower, secondary, university or higher), social class based on occupation (managers, technicians, skilled manual/non manual, semi-skilled/unskilled, homemakers, others), and nationality (Spain, Europe, Central and South America, Africa, Asia), maternal parity (0, 1, 2, 3 or more children) and civil status (married, unmarried), and child sex (female, male). We derived season of conception (winter, spring, summer, autumn) based on the date of conception. We linked a small area-level deprivation index to the maternal residential addresses at delivery. This deprivation index, constructed from the 2011 Population and Housing Census of Spain, included 6 indicators: manual and temporary workers, unemployment, insufficient education overall and in young people [16–29 years], and no access to internet. Deprivation levels were calculated for 35,917 census tracts [30]. Census tracts have a size that varies between 500 and 2,000 inhabitants. This index was able to capture both urban and rural variation in deprivation level. Overall, the deprivation index showed a strong decreasing gradient from South-West to North-East regions, with specific deprivation index patterns within the big metropolitan areas. We calculated the level of urbanicity (cities, towns/suburbs, rural areas) at the maternal residential addresses at delivery using the 2014 DEGURBA classification created by the European Commission [31]. We assigned the Spanish NUTS 2 code to the maternal residential addresses at delivery, corresponding to the 16 Autonomous Communities included in the study, to capture regional differences [32]. Finally, we estimated weekly mean temperature at the maternal residential addresses at delivery using data provided by the European Centre for Medium-Range Weather Forecast at an hourly temporal resolution and a spatial resolution of 10 km x 10 km.

### Statistical analysis

We used the expectation-maximization imputation method using the “Amelia” R package v1.8.3 [33] to impute missing data on the potential confounding variables (Table S1). We obtained similar distributions between observed and imputed datasets (Table S2).

Participants included in the PM_10_ (*N* = 3,678,445) and PM_2.5_ (*N* = 1,991,031) analyses had a higher socioeconomic status and a higher proportion of Spanish nationality compared to those not included (Table S3-S4). We applied inverse probability weighting to correct for potential selection bias as a result of only including those participants with exposure and outcome data available, so that results would be representative for the initial population [34]. This was done for the two study populations separately, the one of the PM_10_ analyses and the one of the PM_2.5_ analyses. We selected the variables that were most predictive of the probability of being included in the study to calculate the inverse of the probability of participation for each participant included. The selected variables were parental age and social class, maternal education level, nationality, parity, and civil status, area-level deprivation index, and urbanicity (Table S5). This inverse probability was assigned as two weights for each participant, one for the PM_10_ analysis and one for the PM_2.5_ analyses, and used in all respective analyses.

First, we used linear regression models to estimate the association between pregnancy-average PM exposure and birthweight and birthweight at term. We used logistic regression models to estimate the association between pregnancy-average PM exposure and low birthweight at term, preterm birth, and small for gestational age. After checking the assumptions of the models, we observed a non-linear association of pregnancy-average PM_2.5_ exposure with birthweight and preterm birth, with a point of inflection around 10 µg/m^3^ (Figure S1). For these two associations, we also found that a natural spline model with 2 degrees of freedom provided better goodness-of-fit than the linear model (likelihood ratio test p-value < 0.001). Thus, as follow-up analysis, we stratified the linear regression models of pregnancy-average PM_2.5_ exposure with birthweight and preterm birth below and above this threshold. Further, to assess the potential impact of individual-level lifestyle risk factors not measured in our birth registry, we applied the indirect adjustment method including data on smoking use during pregnancy and pre-pregnancy body mass index from an ancillary cohort, and indirectly adjusted for them in our analysis of birthweight [35]. To do this, we needed: (i) the estimated associations between these two variables and the exposure to each air pollutant after adjusting for the potential confounding variables included in our main model, and (ii) risk estimates of these two variables on birthweight. We estimated the first information from the INMA Project, a population-based birth cohort set up in Spain, using data from the regions of Valencia, Sabadell, Asturias, and Gipuzkoa collected between 2003 and 2008 (*N* = 2,487) [36]. We extracted the second information from the literature [37, 38].

Second, we applied distributed lag models (DLMs) to identify potential windows of susceptibility to PM exposure across pregnancy in the association with birthweight and small for gestational age. DLMs concurrently estimate the exposure-response relationship and the lag-response relationship using a cross-basis, a bidimensional space of functions obtained from integrating two base functions over the lag dimension that represents the exposure-lag-response relationship. This method allows for the estimation of the time trend for the association between the exposure and the outcome by adjusting exposures at other time points, assuming that the changes are smoothly across weeks [39]. We used a penalized spline constraint to estimate the lag-response relationship allowing 10 degrees of freedom [40]. Since we found a non-linear association of pregnancy-average PM_2.5_ exposure with birthweight, we further explored the shape of the association of the weekly PM_2.5_ exposure with birthweight. We found that all weekly exposure-response association were linear and we considered them as linear in the DLMs (Figure S2). The equation of the model can be found in Methods S2. Since complete exposure data for the exact same weeks is required for this method, we included participants born from week 32 onwards (i.e., exposure up to week 31) in the main analyses (*N* = 3,646,482 for PM_10_ and *N* = 1,973,883 for PM_2.5_) which excluded extreme and very preterm births [41]. The adjusted cumulative effect estimates for periods of interest were calculated by aggregating effect estimates across adjacent lags. As sensitivity analysis, we repeated the models: (i) including participants born from week 28 onwards (i.e., exposure up to week 27) (*N* = 3,670,012 for PM_10_ and *N* = 1,987,222 for PM_2.5_) to investigate a possible selection bias when excluding very preterm births [41], and (ii) including participants born from week 36 onwards (i.e., exposure up to week 35) (*N* = 3,468,035 for PM_10_ and *N* = 1,880,547 for PM_2.5_) to investigate a possible selection bias when excluding all preterm births [41].

Third, all previous analyses were stratified by maternal educational level, area-level deprivation index (categorized in tertiles as low [<-0.687], moderate [-0.687 to 0.126], high [> 0.126]), and the combination of both variables in 4 groups (high education and low deprivation, high education and high deprivation, low education and low deprivation, low education and high deprivation) to investigate whether socioeconomic inequalities were present.

As additional sensitivity analyses, we stratified all analysis by level of urbanicity of the maternal address since population in urban and rural areas might have different individual characteristics and be exposed to PM with different composition. Moreover, we investigated the association of PM_10_ and PM_2.5_ with the gestational-age-adjusted birthweight z-score. Finally, we reran all models adjusting for child sex.

All statistical models were adjusted for the potential confounders described in the previous section. Pregnancy-average temperature across pregnancy was included in the regression models. Weekly temperature across pregnancy was included in the distributed lag models using a natural cubic spline in both the exposure and lag dimension, with internal knots placed at the 25th and 75th percentiles of the temperature distribution and a knot at lag 50th percentile for the lag-response function. We corrected for multiple testing by determining the eigenvalues to identify the effective number of tests using the ‘poolr’ package and ‘meff’ function in R [42]. The effective number of tests was three for the analyses of the pregnancy-average PM (two exposures and five birth outcomes) and one for the analyses of the weekly exposures to PM (two exposures and two birth outcomes) making the new statistical significance level 0.05/3 = 0.017 for the pregnancy-average PM analyses, and 0.05/1 = 0.05 for the weekly PM analyses. All analyses were carried out with R version 4.2.1 [43].

## Results

### Descriptive analysis

Table 1 presents the characteristics of the study population. The mean age of pregnant women was 32 years, and that of their partner was 34 years. The majority of women had attained a secondary educational level (52%), and over half of the households were located in cities (56%). Mean concentrations of PM_10_ and PM_2.5_ during pregnancy were 25.1 µg/m^3^ and 12.7 µg/m^3^, respectively (Table 1, Table S6, Figure S3). PM_10_ and PM_2.5_ were highly correlated (Pearson’s *r* = 0.85). A total of 89% of women were exposed to pregnancy-average PM_2.5_ concentrations above 10 µg/m^3^. Compared to those exposed to concentrations below 10 µg/m^3^, these women more frequently had a slightly lower educational level, a slightly higher likelihood of being born outside Europe, and were more likely to reside in cities (Table S7). The distribution of birth outcomes is shown in Table S8. Mean birthweight was 3248 g and the prevalence of preterm birth was 5.7%. Correlations between birth outcomes ranged from low to moderate (Table S9).

**Table 1:** Characteristics of the study population (*n*=3,678,445)

	Distribution (Percentage or mean (SD))	
*Maternal characteristics*		
Age (years)	31.8	(5.2)
*Educational level*		
High	37.2	
Medium	51.8	
Low	11.0	
*Social class based on occupation*		
Managers	3.1	
Technicians	24.8	
Skilled manual/non-manual	43.0	
Semi-skilled/unskilled	6.7	
Homemakers	20.4	
Others	2.1	
*Region of nationality*		
Spain	89.4	
Europe	3.5	
Central and South America	3.6	
Africa	2.9	
Asia	0.6	
Civil status (married vs. no-married)	70.1	
*Parity*		
No children	54.9	
1 child	36.5	
2 children	6.8	
3 or more children	1.9	
*Paternal characteristics*		
Age (years)	34.2	(5.6)
*Educational level*		
University or higher	26.5	
Secondary	60.0	
Primary or lower	13.5	
*Social class based on occupation*		
Managers	5.3	
Technicians	24.2	
Skilled manual/non-manual	40.0	
Semi-skilled/unskilled	27.0	
Others	3.5	
*Household characteristics*		
Area-level deprivation index^a^	-0.21	(0.99)
*Urbanicity*		
Cities	55.9	
Towns or suburbs	33.9	
Rural areas	10.2	
Mean temperature across pregnancy	16.2	(2.9)
*Particle matter concentrations (µg/m* ^*3*^ *)*		
PM_10_	25.1	(6.6)
PM_2.5_	12.7	(2.4)

*PM*_*10*_ particular matter with aerodynamic diameter less than 10 μm, *PM*_2.5_ particular matter with aerodynamic diameter less than 2.5 μm

^a^Range -2.6 to 4.9

### Pregnancy-average exposure associations

Higher pregnancy-average PM_10_ concentrations were associated with lower birthweight, lower birthweight at term, and an increased odds of preterm birth (e.g., -7.1 g in birthweight [95% Confidence Interval (CI) -8.5; -5.7] and OR 1.04 for preterm birth [95%CI 1.02; 1.05] per 10 µg/m^3^ increase in PM_10_) (Table 2). Higher pregnancy-average PM_2.5_ concentrations were associated with lower birthweight and an increased odds of preterm birth (-3.8 g in birthweight [95%CI -5.9; -1.7] and OR 1.04 for preterm birth [95%CI 1.02, 1.06] per 5 µg/m^3^ increase in PM_2.5_). When pregnancy-average PM_2.5_ concentrations were stratified by 10 µg/m^3^, we only observed an association with birthweight and preterm birth for PM_2.5_ concentrations above 10 µg/m^3^ (-5.4 g in birthweight [95%CI -7.7; -3.1] and OR 1.06 for preterm birth [95%CI 1.04, 1.09] per 5 µg/m^3^ increase in PM_2.5_) compared to PM_2.5_ concentrations below 10 µg/m^3^ (1.9 g in birthweight [95%CI -12.9; 16.7] and OR 0.90 for preterm birth [95%CI 0.79, 1.03] per 5 µg/m^3^ increase in PM_2.5_) (Table S10). When we applied indirect adjustment for smoking use during pregnancy and pre-pregnancy body mass index, we found similar results when indirectly adjusting for body mass index, while effect estimates were slightly stronger and confidence intervals became wider when indirectly adjusting for smoking (Table S11). When comparing the analytical cohort with the ancillary cohort used for the indirect adjustment, we observed that the analytical cohort had a lower proportion of population from a lower socioeconomic position (e.g., low maternal and paternal educational level, semiskilled/unskilled occupations) and also included more population from towns/suburbs and rural areas (Table S12).

When we stratified by maternal educational level, associations of PM_10_ with birthweight and birthweight at term were stronger in infants born to mothers with lower education compared to those with higher education (e.g., -14.3 g in birthweight [95%CI -18.5; -10.2] vs. -4.1 g (95%CI -6.5; -1.8) per 10 µg/m^3^ increase in PM_10_), with non-overlapping confidence intervals (Table 2). No differences across maternal education level strata were observed for preterm birth. Similar patterns were observed for PM_2.5_.

When we stratified by area-level deprivation index, we did not observe differences across strata for either PM_10_ or PM_2.5_, with overlapping confidence intervals for the associations observed in infants born to mothers residing in areas with low deprivation index and those with high deprivation index (Table 2 and S10).

When combining maternal educational level and area-level deprivation index categories, we then observed stronger associations of PM_10_ concentrations with birthweight, birthweight at term, and low birthweight at term in infants born to mothers with both lower education and residing in areas with high deprivation index compared to those with both higher education and residing in areas with low deprivation index (Table 2). Differences between these strata were only observed for PM_2.5_ concentrations in relation to birthweight.

**Table 2:** Adjusted association between pregnancy-average particle matter concentrations and birth outcomes, overall and according to maternal educational level and to area-level deprivation index

	Birthweight		Birthweight at term		Low birthweight at term		Preterm birth		Small for gestational age	
	B	(95% CI)	B	(95% CI)	OR	(95% CI)	OR	(95% CI)	OR	(95% CI)
PM_10_ (∆ 10 μg/m^3^)	(*N*=3,678,445)		(*N*=3,468,035)		(*N*=3,468,035)		(*N*=3,678,445)		(*N*=3,678,445)	
Overall association	**-7.1**	**( -8.5**, -5.7)*	**-4.9**	**( -6.1**, -3.7)*	1.01	(0.99, 1.03)	**1.04**	**(1.02**, 1.05)*	**1.01**	**(1.00**,** 1.02)**
*By maternal educational level*										
High	**-4.1**	**( -6.5**, -1.8)*	**-2.9**	**( -5.0**, -0.7)*	0.99	(0.96, 1.02)	**1.03**	**(1.01**, 1.05)*	**1.02**	**(1.01**, 1.04)*
Medium	**-7.1**	**( -8.9**, -5.2)*	**-4.7**	**( -6.4**, -3.1)*	1.01	(0.99, 1.03)	**1.03**	**(1.02**, 1.07)*	1.00	(0.99, 1.01)
Low	**-14.3**	**(-18.5**, -10.2)*	**-10.4**	**(-14.1**, -6.6)*	**1.05**	**(1.01**,** 1.10)**	**1.06**	**(1.03**, 1.12)*	1.01	(0.98, 1.03)
*By area-level deprivation index*										
Low	**-3.5**	**( -5.8**, -1.2)*	-2.0	( -4.1, 0.1)	0.99	(0.96, 1.02)	**1.02**	**(1.00**,** 1.04)**	1.01	(0.99, 1.03)
Moderate	**-9.9**	**(-12.3**, -7.5)*	**-7.5**	**( -9.7**, -5.4)*	1.00	(0.97, 1.03)	**1.04**	**(1.02**, 1.06)*	1.02	(1.00, 1.03)
High	**-8.4**	**(-10.6**, -3.0)*	**-5.5**	**( -7.7**, -3.3)*	**1.04**	**(1.01**,** 1.07)**	**1.06**	**(1.04**, 1.08)*	1.00	(0.99, 1.02)
*By maternal educational level & area-level deprivation index*										
High education/ Low deprivation	-2.2	( -5.5, 3.7)	-0.1	(-3.1, 2.9)	0.97	(0.92, 1.01)	**1.04**	**(1.01**, 1.08)*	1.01	(0.98, 1.03)
High education/High deprivation	-2.1	( -9.5, 5.8)	-2.0	(-6.8, 2.8)	1.05	(0.98, 1.12)	1.02	(0.97, 1.07)	0.99	(0.95, 1.02)
Low education/Low deprivation	-11.5	(-17.8, 17.6)	**-9.0**	**(-17.3**,** -0.7)**	1.02	(0.91, 1.13)	1.04	(0.97, 1.12)	1.04	(0.98, 1.10)
Low education/High deprivation	**-16.2**	**(-25.7**, -7.1)*	**-11.4**	**(-16.7**, -6.1)*	**1.08**	**(1.02**, 1.14)*	**1.08**	**(1.04**, 1.12)*	1.02	(0.99, 1.06)

	Birthweight		Birthweight at term		Low birthweight at term		Preterm birth		Small for gestational age	
	B	(95% CI)	B	(95% CI)	OR	(95% CI)	OR	(95% CI)	OR	(95% CI)
**PM**_**2.5**_ **(∆ 5 μg/m**^**3**^**)**	(*N*=1,991,031)		(*N*=1,880,547)		(*N*=1,880,547)		(*N*=1,991,031)		(*N*=1,991,031)	
Overall association	**-3.8**	**( -5.9**, -1.7)*	-1.4	( -3.3, 0.4)	0.99	(0.96, 1.01)	**1.04**	**(1.02**, 1.06)*	1.01	(0.99, 1.02)
By maternal educational level										
High	-1.7	( -5.0, 1.6)	-0.1	( -3.0, 2.8)	0.97	(0.92, 1.01)	**1.05**	**(1.01**, 1.08)*	0.99	(0.97, 1.02)
Medium	-3.0	( -6.1, 0.0)	-0.9	( -3.6, 1.7)	0.99	(0.95, 1.02)	**1.03**	**(1.01**,** 1.06)**	1.01	(0.99, 1.03)
Low	**-13.1**	**(-19.9**, -6.3)*	**-7.0**	**(-13.0**,** -1.0)**	1.03	(0.96, 1.10)	**1.08**	**(1.03**, 1.13)*	1.01	(0.97, 1.05)
By area-level deprivation index										
Low	0.1	( -3.4, 3.6)	0.5	( -2.6, 3.7)	0.96	(0.92, 1.01)	1.01	(0.98, 1.05)	1.01	(0.98, 1.03)
Moderate	**-6.3**	**( -9.0**, -2.6)*	-2.9	( -6.1, 0.3)	0.99	(0.94, 1.03)	**1.06**	**(1.03**, 1.09)*	1.00	(0.98, 1.03)
High	**-6.7**	**(-10.6**, -2.8)*	-2.9	( -6.3, 0.6)	1.02	(0.97, 1.06)	**1.06**	**(1.03**, 1.10)*	1.01	(0.99, 1.04)
By maternal educational level & area-level deprivation index										
High education/low deprivation	-0.9	( -5.5, 3.7)	0.4	( -3.7, 4.6)	0.94	(0.88, 1.01)	1.05	(1.00, 1.09)	0.99	(0.95, 1.02)
High education/high deprivation	-1.9	( -9.5, 5.8)	1.2	( -5.6, 8.0)	1.03	(0.93, 1.13)	**1.10**	**(1.03**, 1.17)*	1.00	(0.95, 1.06)
Low education/low deprivation	-0.1	(-17.8, 17.6)	-7.0	(-22.5, 8.5)	0.97	(0.79, 1.15)	1.03	(0.89, 1.17)	1.01	(1.01, 1.12)
Low education/high deprivation	**-16.4**	**(-25.7**, -7.1)*	-8.0	(-16.2, 0.3)	1.05	(0.96, 1.15)	**1.11**	**(1.04**, 1.18)*	1.01	(0.96, 1.06)

*B* Beta coefficient, *CI* Confidence interval, *OR* odds ratio, *PM*_*10*_ particular matter with aerodynamic diameter less than 10 μm, *PM*_2.5_ particular matter with aerodynamic diameter less than 2.5 μm

Adjusted for parental age, parental educational level, parental social class based on occupation, maternal nationality, maternal civil status, parity, area-level deprivation index, urbanicity, month and year of conception, mean temperature across pregnancy, and geographical region. Stratified models were not adjusted for the stratification variable (i.e., maternal educational level and/or area-level deprivation index)

In bold, associations with a p-value ≤ 0.05. *Associations that survive correction for multiple testing (p-value ≤ 0.017)

### Windows of susceptibility

**Fig. 2:**
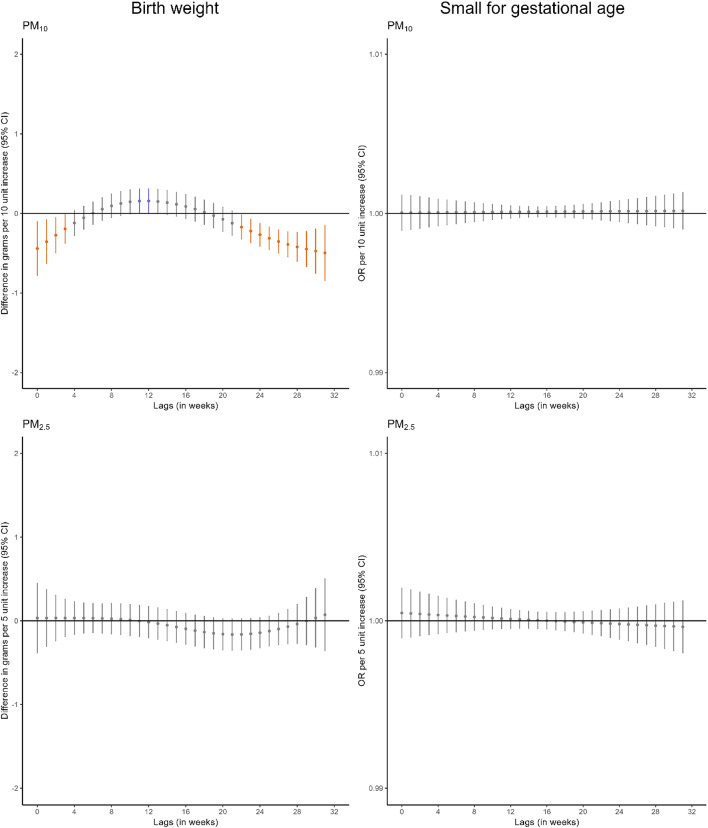
Adjusted lag-response association of weekly-average particle matter concentrations during pregnancy with birthweight and small for gestational age. *CI* confidence interval, *OR* odds ratio, *PM*_*10*_ particular matter with aerodynamic diameter less than 10 μm, *PM*_*2*.5_ particular matter with aerodynamic diameter less than 2.5 μm. Adjusted for parental age, parental educational level, parental social class based on occupation, maternal nationality, maternal civil status, parity, area-level deprivation index, urbanicity, month and year of conception, weekly temperature across pregnancy, and geographical region. Dots represent the effect estimates of the association between the exposure at each specific lag and the outcome. Vertical gray, blue, and orange lines represent 95% CI and indicate no divergence from the null, significant divergence from positive association, and significant divergence from negative association, respectively. All associations survived correction for multiple testing (p-value ≤ 0.05)

We identified some windows of susceptibility to PM_10_ in relation to birthweight (Fig. 2, Table S13). Higher PM_10_ exposure during the first 4 weeks of pregnancy was associated with lower birthweight (-1.3 g [95%CI -2.3; -0.3] per 10 µg/m^3^ increase in PM_10_). Also, higher PM_10_ exposure between weeks 23 and 32 of pregnancy was associated with lower birthweight (-3.6 g [95%CI -4.9; -2.2] per 10 µg/m^3^ increase in PM_10_). However, we did not identify windows of susceptibility to PM_10_ in relation to small for gestational age or to PM_2.5_ in relation to either birthweight or small for gestational age. Comparable results were observed when we excluded very preterm births (Figure S4) or all preterm births (Figure S5).

**Fig. 3:**
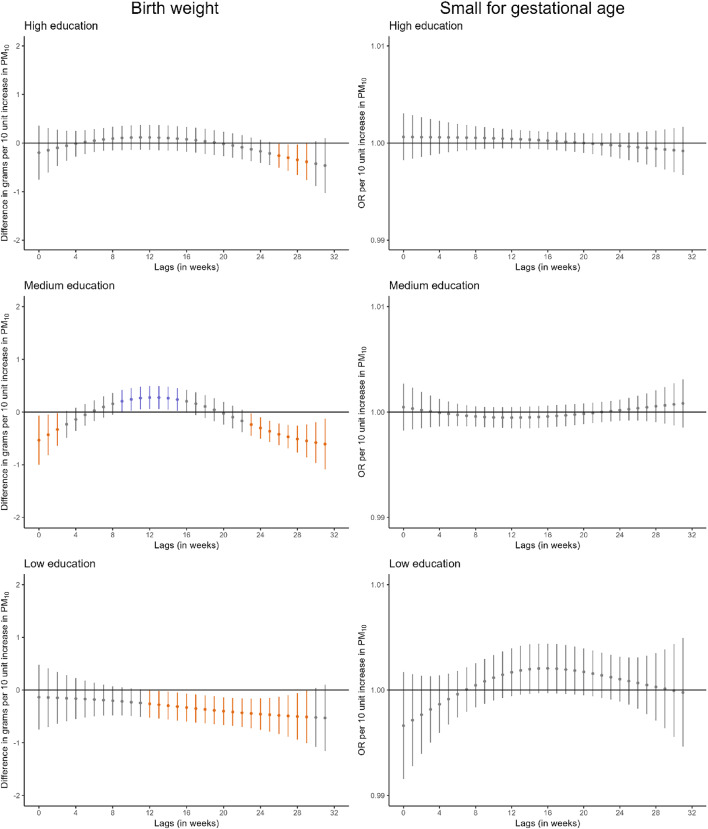

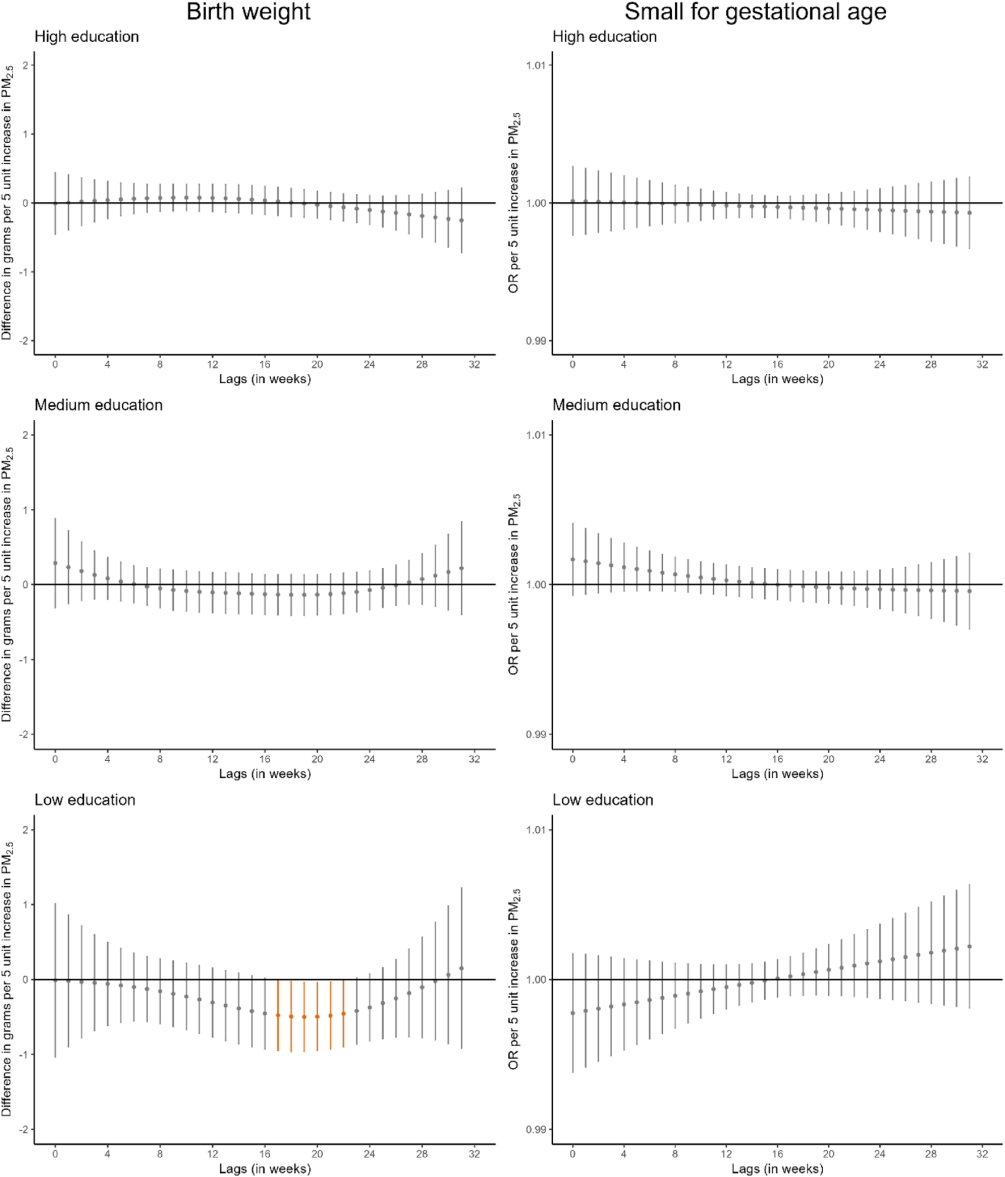
Adjusted lag-response association of weekly-average PM_10_ and PM_2.5_ concentrations during pregnancy with birthweight and small for gestational age according to maternal educational level. *CI*, confidence interval; *OR*, odds ratio; *PM*_*10*_, particular matter with aerodynamic diameter less than 10 μm; *PM*_*2*.5_, particular matter with aerodynamic diameter less than 2.5 μm. Adjusted for parental age, paternal educational level, parental social class based on occupation, maternal nationality, maternal civil status, parity, area-level deprivation index, urbanicity, month and year of conception, weekly temperature across pregnancy, and geographical region. Dots represent the effect estimates of the association between the exposure at each specific lag and the outcome. Vertical gray, blue, and orange lines represent 95% CI and indicate no divergence from the null, significant divergence from positive association, and significant divergence from negative association, respectively. All associations survived correction for multiple testing (p-value ≤ 0.05)

Stratified analyses by maternal educational level suggested that the window of susceptibility to PM_10_ at the end of pregnancy in relation to birthweight was larger in infants born to mothers with lower education (Fig. 3, Table S14). For PM_2.5_, we identified a narrow window of susceptibility between weeks 18 and 23 in relation to birthweight in infants born to mothers with lower education.

**Fig. 4:**
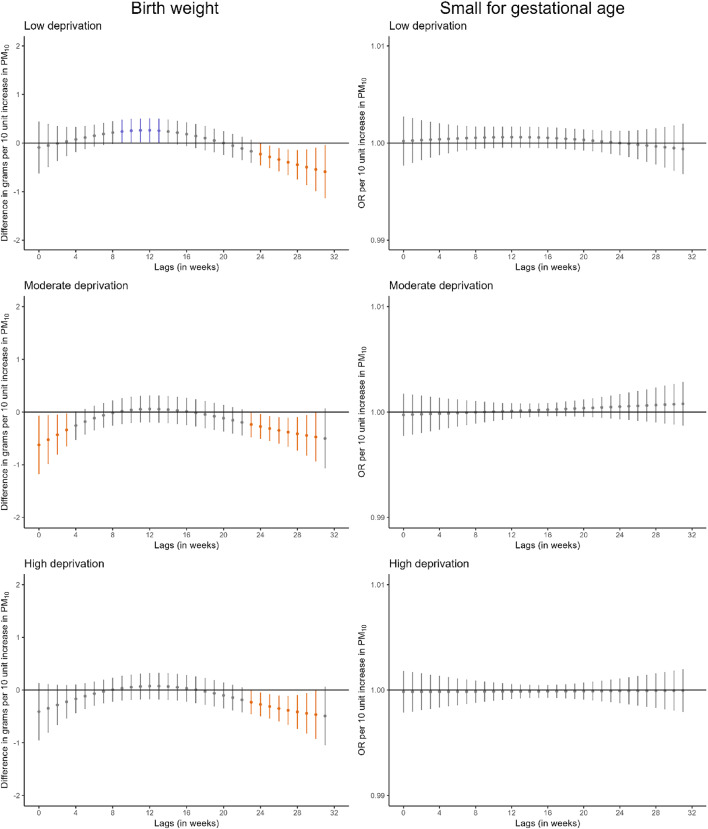

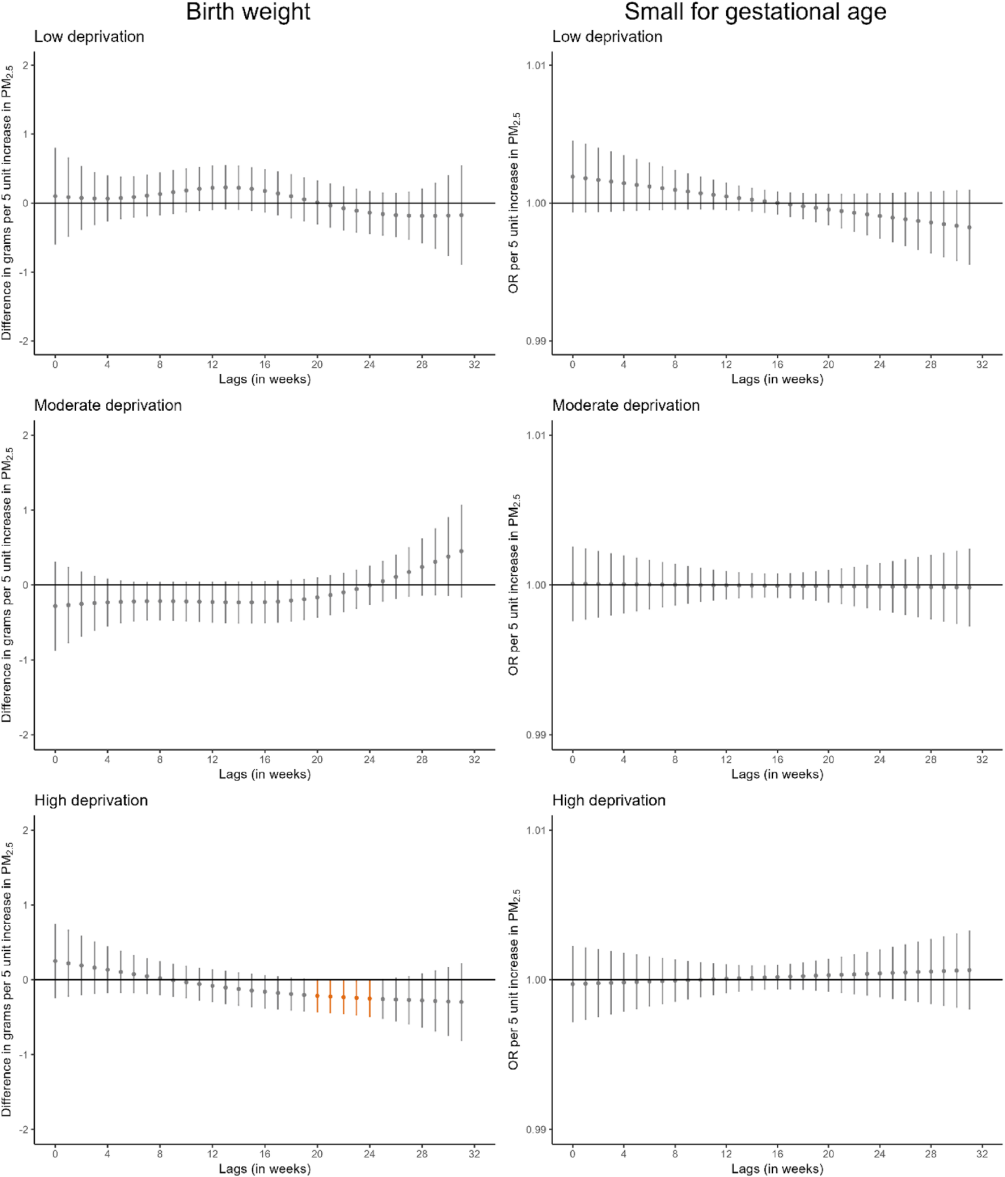
Adjusted lag-response association of weekly-average PM_10_ and PM_2.5_ concentrations during pregnancy with birthweight and small for gestational age according to area-level deprivation index. CI, confidence interval; OR, odds ratio; PM_10_, particular matter with aerodynamic diameter less than 10 μm; PM_2.5_, particular matter with aerodynamic diameter less than 2.5 μm. Adjusted for parental age, parental educational level, parental social class based on occupation, maternal nationality, maternal civil status, parity, urbanicity, month and year of conception, weekly temperature across pregnancy, and geographical region. Dots represent the effect estimates of the association between the exposure at each specific lag and the outcome. Vertical gray, blue, and orange lines represent 95% CI and indicate no divergence from the null, significant divergence from positive association, and significant divergence from negative association, respectively. All associations survived correction for multiple testing (p-value ≤ 0.05)

When we stratified the analyses by area-level deprivation index, the window of susceptibility to PM_10_ at the end of pregnancy in relation to birthweight appeared consistent across strata (Fig. 4, Table S14). For PM_2.5_, a narrow window of susceptibility between weeks 21 and 25 was identified in relation to birthweight in infants born to mothers residing in areas of high deprivation index.

Additionally, a large window of susceptibility to PM_10_ at the end of pregnancy in relation to birthweight was identified in infants born to mothers with both lower education and residing in areas of high deprivation index, and in mid pregnancy in infants born to mothers with both lower education and residing in areas of low deprivation index (Figure S6, Table S15). For PM_2.5_, the narrow window of susceptibility between weeks 20 and 26 in relation to birthweight was identified only in infants born to mothers with both lower education and residing in areas of low deprivation index.

### Additional sensitivity analysis

Stratifying by level of urbanicity of the maternal residential address at delivery did not reveal consistent patterns across strata (Table S16-S17, Figure S7). Moreover, analysis of gestational-age-adjusted birthweight z-score showed that higher pregnancy-average PM_10_ concentrations were associated with a lower birthweight score (-0.004 (95%CI -0.007, -0.001) per 10 µg/m^3^ increase in PM_10_), whereas no association was observed with pregnancy-average PM_2.5_ concentrations (0.003 (95%CI -0.002, 0.007) per 5 µg/m^3^ increase in PM_2.5_) (Table S18). Finally, effect estimates of pregnancy-average PM_10_ concentrations remain almost identical when we adjusted for child sex, while effect estimates of PM_2.5_ concentrations moved slightly toward the null (Table S19, Figure S8).

## Discussion

In this large, population-based study using national birth registry data from Spain, we observed that exposure to higher concentrations of both PM_10_ and PM_2.5_ during pregnancy was associated with adverse birth outcomes, including lower birthweight and increased odds for preterm birth. These associations varied by socioeconomic indicators at both individual and area levels, with overall stronger associations among infants born to mothers with lower educational attainment or those with both lower educational attainment and residing in more deprived areas. Further, the third trimester emerged as the period of higher susceptibility to PM_10_ exposure, with a pattern of effect modification by socioeconomic indicators consistent with that observed for pregnancy-average exposure. In contract, we did not identify windows of higher susceptibility to PM_2.5_.

Extensive epidemiological evidence has provided support for an association between pregnancy-average air pollution exposure, in particular PM_2.5_, and adverse birth outcomes [4–14]. In our study, we observed larger effect estimates for PM_10_ compared to PM_2.5_, and a window of susceptibility was only identified for PM_10_. These findings were unexpected, given that smaller particles are generally considered more harmful due to their greater capacity for deep lung penetration and systemic inflammation [4, 5]. Of note, our results suggest that the association between PM_2.5_ and birthweight may be largely attributable to reduced gestational duration (i.e., results were stronger for preterm birth or birthweight compared to birthweight at term or small for gestational age, and no association was observed with gestational-age-adjusted birthweight z-score). Thus, PM_2.5_ effects seem to be more related to gestational age than to growth restriction of the fetus. In contrast, associations with PM_10_ appeared to extend beyond gestational duration. These findings differ from some earlier studies that reported stronger associations with fetal growth restriction [5], although direct comparisons are limited as most studies did not examine these outcomes in parallel [4, 6–9]. Furthermore, most of our pregnant women were exposed to air pollution concentrations below the EU regulatory limits in place at the time of the study (40 µg/m^3^ for PM_10_ and 25 µg/m^3^ for PM_2.5_). Our findings provide additional evidence on the need to lower these limits as indicated in the new WHO air quality guidelines of 2021 [3] and by the new EU air quality standards [44].

Most previous studies assessed average air pollution exposure over pregnancy or within clinically defined trimesters. These approaches limit the ability to detect susceptible periods of exposure, which are essential for designing targeted interventions. The fetal period comprises a series of dynamic developmental phases, each potentially sensitive to environmental insults depending on the timing, duration, and intensity of exposure. Some recent studies employed temporally resolved exposure models to examine daily or weekly PM concentrations in relation to birthweight and small for gestational age, leading to heterogeneous results [15–21]. Most studies identified the second and/or third trimesters (between weeks 13 and 39, depending on the study) as susceptible periods for PM_2.5_ exposure in relation to lower birthweight [15–19], though some also observed earlier exposures, including the preconception period and the first trimester (between 12 weeks before conception and the week 13 of pregnancy) [20], or found no susceptible periods [21]. For PM_10_, susceptibility during the third trimester (between weeks 22 and 30) has been reported in relation to lower birthweight [19]. Fewer studies investigated these associations with small for gestational age, showing inconsistent findings [18, 20, 21]. Our findings corroborate the third trimester as a key period of susceptible to PM_10_ in relation to birthweight. Little is known on the differential biological pathways of PM exposure at different timings of pregnancy. While the first trimester is crucial for organogenesis, fetal growth and maturation takes place mostly in the last two trimesters of pregnancy when oxygen and nutrients demand increase substantially [45]. Plausible biological mechanisms for the observed associations include placental dysfunction, oxidative stress, inflammation, and epigenetic changes [5], which may exert more pronounced effects in the second half of pregnancy. Our findings underscore the importance of targeted public health interventions to minimize PM exposure, particularly during the latter stages of pregnancy.

A limited number of studies have explored the role of socioeconomic inequalities in the association between pregnancy-average PM exposure and birth outcomes. Most have focused on race and ethnic disparities, with evidence suggesting greater vulnerability among infants born to African-American or Black mothers [24, 25]. Our study contributes additional evidence demonstrating stronger associations in infants born to mothers with lower educational attainment, in particular when combined with residence in more deprived areas. Although prior previous research has suggested similar patterns [24, 25], limited sample sizes and challenges in harmonizing educational attainment categories across studies have hindered robust conclusions. In addition, one study examining employment status as a proxy for socioeconomic position reported no differential effects on low birthweight or small for gestational age [46]. Studies that attempted to identify critical windows of susceptibility of PM exposure on adverse birth outcomes across different individual or area-level socioeconomic inequality strata yielded mixed results [18, 26, 27]. One study identified early- to mid-pregnancy as susceptible windows to PM_2.5_ and PM_10_ exposure in relation to lower birthweight among pregnant women residing in areas with higher neighborhood-level cumulative burden [27], while others found that PM_2.5_ exposure from weeks 7 and 11 and from weeks 27 and 37 was related to lower birthweight in pregnant women residing in areas with lower neighborhood-level vulnerability [26], or observed no differential effects [18]. Further research is warranted to better understand the role of socioeconomic inequalities on the association between air pollution and birth outcomes to inform the design of efficient equitable interventions.

This study has several strengths. It is based on a large, nationwide sample size spanning multiple years, with spatially and temporally resolved PM exposure estimates derived from a standardized and validated methodology, and the inclusion of spatial confounding indicators. The availability of both individual- and area-level socioeconomic indicators allowed for a comprehensive analysis of socioeconomic inequalities. The use of DLM enabled identification of susceptible periods of exposure without relying on predefining arbitrary periods, while minimizing issues related to multiple testing due to the concurrent estimation of the lag-exposure-outcome association.

There are some limitations that should be considered. First, PM concentrations were estimated based on the maternal residential address at delivery, assuming no residential mobility during pregnancy. Although previous data suggested low mobility in a previous birth cohort study in Spain (i.e., 6%) [47, 36], pregnant women that moved might have different socioeconomic characteristics which would lead to a differential misclassification error. This could affect the results, in particular in the stratified analysis by socioeconomic inequalities, in which effect estimates could be biased either toward or away from the null. In addition, we could not account for individual commuting patterns, or locations where pregnant women could have spent their time (e.g., at work). This may have resulted in exposure misclassification, although a previous study in Spanish pregnant women showed almost no change in nitrogen dioxide concentrations when time spent at work was included in the air pollution estimation [48]. Second, we did not have information on lifestyle and behavioral individual variables such as maternal smoking or body mass index. While these are well-known risk factors for adverse birth outcomes, they are not expected to be directly associated with air pollution exposure, once contextual socioeconomic variables are adjusted for. It would have been relevant to adjust for them under the assumption of being indirectly related to the exposure as proxies of socioeconomic status, although this is debatable for body mass index that can be considered a potential mediator. In order to overcome this limitation, we applied an indirect adjustment method including data on smoking during pregnancy and body mass index from an ancillary cohort which yielded to similar results. The use of the INMA birth cohort as ancillary cohort, even if it is the largest population-based birth cohort in Spain, could have introduced residual confounding in the indirect adjustment analysis due to the lack of representativeness. However, we do not expect this to be high since we carefully adjusted for area-level confounding. Third, we lacked exposure models for nitrogen dioxide or nitrogen oxides, which are also relevant to birth outcomes [5, 7, 8, 12]. Fourth, the area-level deprivation index was only available for 2011, limiting temporal representativeness. However, spatial patterns of deprivation in Spain might be relatively stable over time, suggesting that this measure remains informative. Fifth, although DLMs offer advantages for identifying time-specific association, they require multiple specifications and assumptions that may influence results. Our main findings remained unchanged after the different sensitivity analysis, but results should be interpreted with caution, in particular the analysis stratifying by maternal educational level where groups become smaller. Lastly, 33% of the initial sample lacked information on the maternal residential address at delivery or on birthweight and gestational age and could not be directly included in the analysis. These participants were disproportionately from lower socioeconomic backgrounds, potentially introducing selection bias. We applied inverse probability weighting to mitigate this bias. However, we might have missed relevant predictors to properly estimate the weights and not have been able to fully eliminate this bias, leading to an underestimation of the true association. This is particularly relevant for the stratified analysis on socioeconomic variables, where the included population of infants born to mothers with lower levels of education and residing in more deprived areas was under represented.

In conclusion, we found that pregnancy-average exposure to PM was associated with low birthweight and preterm birth, with stronger associations among infants of socioeconomically disadvantaged mothers. Our findings also suggest that the third trimester of pregnancy may represent a critical window of susceptibility to PM_10_ in relation to birthweight, and this susceptibility might be also more pronounced in socioeconomically disadvantaged populations. These findings underscore the need for structural policies to reduce current PM levels in pregnancy women and socioeconomic inequalities. Future research should continue to investigate the role of socioeconomic inequalities on the exposure to PM to inform equitable and effective public health interventions.

## Electronic supplementary material

Below is the link to the electronic supplementary material.

Supplementary Material 1
