# Supplementary material for: Particulate matter exposure during pregnancy and birth outcomes: exposure windows of susceptibility and socioeconomic inequalities
Source: Eur J Epidemiol. 2025 Jul 24;40(9):1105–21. doi: 10.1007/s10654-025-01274-1 (PMC12537619; doi:10.1007/s10654-025-01274-1)
Supplement: Supplementary file 1 — Supplementary Material 1 [file 10654_2025_1274_MOESM1_ESM.docx]

**Particulate matter exposure during pregnancy and birth outcomes: exposure windows of susceptibility and socioeconomic inequalities**

Mònica Guxens, Núria Botella, Massimo Stafoggia, Marcelle Canto, Sami Petricola, Antònia Valentín, Aitana Lertxundi, Ana Fernández-Somoano, Carmen Freire, Anna García-Altés, Elia Diez, Marc Marí-Dell’Olmo, Carmen Iñiguez, María José López, Rebeca Ramis, Anne-Claire Binter

**Table of contents**

[Methods S1. Particulate matter modelling 3](#_Toc201109104)

[Table S1. Details of the imputation modelling 7](#_Toc201109105)

[Table S2. Comparison of the observed and imputed data and the percentage of missing data 8](#_Toc201109106)

[Table S3. Characteristics of the participants included and not included in the PM_10_ analysis 9](#_Toc201109107)

[Table S4. Characteristics of the participants included and not included in the PM_2.5_ analysis 11](#_Toc201109108)

[Table S5. List of variables explored to calculate the inverse probability of attrition weights and those included in the analysis 13](#_Toc201109109)

[Figure S1. Shape of the association between pregnancy average particulate matter concentrations and birth outcomes 14](#_Toc201109110)

[Figure S2. Shape of the association of PM_2.5_ exposure at weeks 1, 5, 10, 15, 20, 25, 30, and 32 with birthweight 17](#_Toc201109111)

[Methods S2. Equation of the distributed lag model 19](#_Toc201109112)

[Table S6. Distribution of particle matter concentrations during pregnancy 20](#_Toc201109113)

[Figure S3. Distribution of particle matter concentrations during pregnancy 21](#_Toc201109114)

[Table S7. Characteristics of the participants with pregnancy-average PM_2.5_ levels below and above 10 μg/m^3^ 22](#_Toc201109115)

[Table S8. Distribution of birth outcomes 23](#_Toc201109116)

[Table S9. Spearman correlation between birth outcomes 24](#_Toc201109117)

[Table S10. Adjusted association of pregnancy-average PM_2.5_ concentrations with birthweight and preterm birth below and above 10 μg/m^3^, overall and according to maternal educational level and to area-level deprivation index 25](#_Toc201109118)

[Table S11. Adjusted association of pregnancy-average PM_10_ and PM_2.5_ concentrations with birthweight after applying indirect adjustment for smoking use during pregnancy and pre-pregnancy body mass index 27](#_Toc201109119)

[Table S12. Characteristics of the analytical and ancillary cohort 28](#_Toc201109120)

[Table S13. Adjusted lag-response association of weekly-average PM_10_ and PM_2.5_ concentrations with birthweight and small for gestational age according to maternal educational level and area-level deprivation index 29](#_Toc201109121)

[Figure S4. Adjusted lag-response association of weekly-average PM_10_ and PM_2.5_ concentrations during pregnancy with birthweight and small for gestational age including all participants born from week 28 onwards 30](#_Toc201109122)

[Figure S5. Adjusted lag-response association of weekly-average PM_10_ and PM_2.5_ concentrations during pregnancy with birthweight and small for gestational age including all participants born from week 36 onwards 31](#_Toc201109123)

[Table S14. Adjusted lag-response association of weekly-average PM_10_ and PM_2.5_ concentrations with birthweight and small for gestational age according to maternal educational level and area-level deprivation index 32](#_Toc201109124)

[Figure S6. Adjusted lag-response association of weekly-average PM_10_ and PM_2.5_ concentrations during pregnancy with birthweight and small for gestational age according to the combination of maternal educational level and area-level deprivation index 33](#_Toc201109125)

[Table S15. Adjusted lag-response association of weekly-average PM_10_ and PM_2.5_ concentrations with birthweight and small for gestational age according the combination of maternal educational level and area-level deprivation index 35](#_Toc201109126)

[Table S16. Adjusted association between pregnancy-average particle matter concentrations and birth outcomes, overall and according to level of urbanicity of the maternal address 36](#_Toc201109127)

[Table S17. Adjusted association of pregnancy-average PM_2.5_ concentrations with birthweight and preterm birth below and above 10 μg/m^3^, overall and according to level of urbanicity of the maternal address 37](#_Toc201109128)

[Figure S7. Adjusted lag-response association of weekly-average PM_10_ and PM_2.5_ concentrations during pregnancy with birthweight and small for gestational age according to level of urbanicity of the maternal address 38](#_Toc201109129)

[Table S18. Adjusted association of pregnancy-average PM_10_ and PM_2.5_ concentrations with gestational-age-adjusted birthweight z-score 40](#_Toc201109130)

[Table S19. Adjusted association between pregnancy-average particle matter concentrations and birth outcomes additionally adjusted for child sex 41](#_Toc201109131)

[Figure S8. Adjusted lag-response association of weekly-average particle matter concentrations during pregnancy with birthweight and small for gestational age additionally adjusted for child sex 42](#_Toc201109132)

[References 43](#_Toc201109133)

# **Methods S1. Particulate matter modelling**

***Study area***

Spain is located in Southwestern Europe, being the largest country in Southern Europe and the fourth-most populous European Union member state. The study area included the peninsular Spain, spanning across the majority of the Iberian Peninsula, and the Balearic Islands in the Mediterranean Sea. The Canary Islands in the Atlantic Ocean and the autonomous cities of Ceuta and Melilla in North Africa were not included. The [peninsular](https://en.wikipedia.org/wiki/Peninsular_Spain) Spain covers an area of about 500,000 km^2^. We divided the study area into 1km x 1km grid cells, with a total of 505,685 grid cells.

***Particulate matter data***

We retrieved daily and hourly particulate matter (PM) concentrations from the national air quality database of the *Ministerio para la Transición Ecológica y el Reto Demográfico* between January 2003 and December 2016 for PM_10_ and between January 2009 and December 2016 for PM_2.5_. The number of monitoring sites increased across the years (i.e., from 204 in 2003 to 413 in 2016 for PM_10_ and from 176 in 2009 to 211 in 2016 for PM_2.5_) (Figure A).


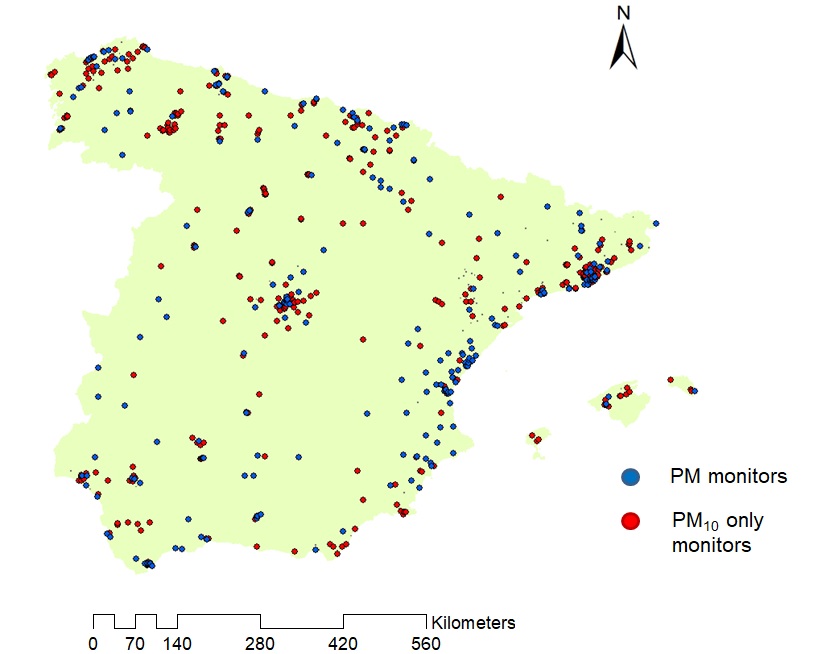


**Figure A. Spatial distribution of the monitoring stations from 2003 to 2016 in Spain**

***Spatiotemporal predictors***

We selected several spatiotemporal predictors that had high spatial and temporal resolution over the study area and estimated them for the entire study period between 2003 and 2016.

Aerosol Optical Depth (AOD). AOD is a quantitative estimate of the number of aerosols present in the atmosphere, and it can be used as a proxy for surface particles. We used the Multi-Angle Implementation of Atmospheric Correction (MAIAC) algorithm developed by NASA that provides daily AOD data at a spatial resolution of 1km x 1km at 2 different wavelengths (470 and 550 nm). Similar to previous works, we used MAIAC AOD based on MODIS product MCD19A2 Version 6 [1] for the years 2003-2016. Furthermore, since MAIAC AOD was missing on a large number of days and grid cells due to cloud coverage, water/snow glint reflectance, and satellite calibration, we also used AOD data from the Monitoring Atmospheric Composition and Climate – Interim Implementation (MACC-II) project developed within the Copernicus Atmosphere Monitoring Service (CAMS) and provided by the European Centre for Medium-Range Weather Forecasts (ECMWF) website [2]. We included daily total AOD at 5 different wavelengths (469, 550, 670, 865, and 1240 nm) at a spatial resolution of 0.75º x 0.75º (111km x 85km approximately at Spanish latitude).

Meteorological data. We included hourly mean air temperature at 2 meters, planetary boundary layer height, dew point at 2 meters, sea-level barometric pressure and total cloud coverage at 0h and 12h, daily total precipitations, and average wind speed and direction at 10 meters from the ERA-Interim reanalysis project [3] at a spatial resolution of 0.25º x 0.25º (28km x 20km approximately at Spanish latitude) at noon and at midnight of each day.

Normalized Difference Vegetation Index (NDVI). We included monthly estimated of NDVI from the MODIS NDVI product (MOD13A3) at a spatial resolution of 1km x 1km.

Saharan dust advections. We defined daily absence or presence of a Saharan dust advection episode at each grid cell using a combination of atmospheric tools [4].

***Spatial predictors***

We selected a number of spatial predictors that had high spatial resolution over the study area and estimated them for the entire study period between 2003 and 2016.

Light at night (LAN). We included LAN for the year 2015 from the Visible Infrared Imaging Radiometer Suite (VIIRS) Day/Night Band (DNB) [5] at a spatial resolution of 30 arc-second (750m x 750m approximately at Spanish latitude).

Impervious surface area (ISA). We included ISA of the year 2012 from the Copernicus Land Monitoring Service (CLMS) at a spatial resolution of 20m x 20m.

Elevation. We included mean elevation of each grid cell using a Digital Elevation Model of 200m pixel resolution (MDT200) from the Spanish National Geographic Institute (IGN).

Roads. We included the Euclidean distance between the grid cell centroid and highways or major roads and aggregated road density at each grid cell by highway, major and secondary roads, and local roads. Road data was obtained from Navteq dataset published by ESRI for the year 2012.

Administrative boundaries. We defined administrative boundaries as autonomous communities using the Spanish National Geographic Institute.

Land cover data. We included the percentage of each grid cell and all neighbor grid cells covered by the 17 classes of Corine Land Cover (CLC) (i.e., high/low development, urban green, industries, arable land, pastures, deciduous/evergreen/forest/shrubs, water). We used CLC data of the year 2000 for the modelling between 2003 and 2005, CLC data of the year 2006 for the modelling between 2006 and 2011, and CLC data of the year 2012 for the modelling between 2012 and 2016.

Slope. We included mean and 90^th^ percentile slope of each grid cell using a Digital Elevation Model of 200m pixel resolution (MDT200) from the Spanish National Geographic Institute.

Population. We included the resident population in each grid cell using the area weighted population of the Spanish census tracts of the year 2001 for the modelling between 2003 and 2010 and of the year 2011 for the modelling between 2011 and 2016.

Climate classification. We included the main and detailed Köppen_Geiger climate classification in each grid cell using the re-analyzed Köppen-Geiger map [6] is provided with a resolution of 5 arc minutes for the more recent 25-year period 1986-2010.

Phytoclimatic types. We included the phytoclimatic classification in each grid cell using the phytoclimatic regions map for Spain published for the year 1990 by the *Ministerio para la Transición Ecológica y el Reto Demográfico*.

***Statistical modelling***

We implemented a 5-stage machine-learning approach, based on a random forest methodology.

Stage 1. We predicted daily PM_2.5_ concentrations in monitors where only PM_10_ concentrations were measured using a random forest model separately for each year between 2009 and 2016. Thus, the main predictive variable was daily PM_10_ concentrations from co-located monitors. Models also included month, day of the week, geographical coordinates of the monitor, type of monitor (i.e., traffic, industrial, background), type of location of the monitor (i.e., urban, suburban, rural), and type of area of the monitor (agricultural, commercial, industrial, residential, etc.). Out-of-bag R^2^ were between 0.78 and 0.84 and 10-fold cross-validated R^2^ were between 0.58 and 0.69.

Stage 2. MAIAC AOD data was missing between 53% in 2005 and 70% in 2014. We imputed missing MAIAC AOD from CAMS AOD estimated using a random forest model separately for each year between 2003 and 2016 and for the 2 wavelengths 470nm and 550nm. Thus, the main predictive variable was daily CAMS AOD at 5 wavelengths (469, 550, 670, 865, and 1240 nm) at 3-hour windows. Models also included day of year and geographical coordinates of the centroid of the grid cell. Out-of-bag R^2^ were between 0.84 and 0.89.

Stage 3. We estimated the association of daily PM concentrations with spatiotemporal and spatial predictors using a random forest model separately for each year between 2003 and 2016 for PM_10_ and between 2009 and 2016 for PM_2.5_ in order to predict PM over grids and days without monitoring stations. We included PM concentrations on the log scale (PM_10_ from the measurements and PM_2.5_ from the Stage 1 estimates). Main predictors were MAIAC AOD estimates from Stage 2, day of the year, month, day of the week, coordinates of the centroid of the grid cell, and all spatiotemporal and spatial predictors detailed above. For PM_10_, out-of-bag R^2^ were between 0.68 and 0.80 and 10-fold cross-validated R^2^ were between 0.46 and 0.56. For PM_2.5_, out-of-bag R^2^ were between 0.68 and 0.78 and 10-fold cross-validated R^2^ were between 0.46 and 0.58.

Stage 4. We obtained estimates of daily mean PM concentrations for each grid cell by applying the stage 3 model fit.

Stage 5. We predicted PM variability within grid cells using small-scale temporal and spatial predictors at the monitor level applying a random forest model separately for each year between 2003 and 2016 for PM_10_ and between 2009 and 2016 for PM_2.5_. Models included as outcome the residuals of the stage 3 10-fold cross-validation estimates (e.g., amount of PM variability not explained by the 1km x 1km predictors). Predictors were month, day of the week, light at night at the monitor, impervious surface area at the monitor, elevation at the monitor, roads within a buffer of 150m around the monitor, and total population within a buffer of 150m around the monitor.

Stage 6. We estimate daily PM concentration at the addresses level available from an epidemiological study. First temporal and spatial predictors defined in Stage 5 are calculated at the addresses level. Then we predict the output of the random forest from Stage 5 to the address level. Finally, we sum this prediction to the prediction of the Stage 3 at the 1km x 1km grid cell where the address is located.

Statistical packages. PostgreSQL 11.17, PostGIS 2.5.5, and R version 4.2.1 were used.

# **Table S1. Details of the imputation modelling**

| **Software used and key setting:** R (version 4.3.2; R Core Team (2023)) – Amelia package v1.8.3 |
| --- |
| **Number of imputed datasets created:** 1 |
| **Variables included in the imputation procedure*:**  child sex, maternal age, maternal country of birth, maternal educational level, maternal social class, maternal parity, paternal age, paternal education level, paternal social class, marital status, deprivation index, urbanicity, season of conception, average temperature across pregnancy, autonomous community, and year of conception.  **Potential predictors for a given variable were restricted to those that had: i) an absolute correlation greater than 0.09 with the given variable; and ii) a proportion of non-missing observations greater than 25% among the observations with missing values in that given variable.* |
| **Diagnostics:** Visual inspection by checking convergence and density plots |
| **References:** (Honaker et al., 2012) |

# **Table S2. Comparison of the observed and imputed data and the percentage of missing data**

|  | **Observed data** | **Imputed data** | **% Missing data** |
| --- | --- | --- | --- |
| **Maternal characteristics** |  |  |  |
| Educational level |  |  | 29.7 |
| High | 37.2 | 33.0 |  |
| Medium | 51.7 | 54.7 |  |
| Low | 11.0 | 12.3 |  |
| Social class based on occupation |  |  | 9.9 |
| Managers | 3.1 | 3.4 |  |
| Technicians | 24.8 | 24.4 |  |
| Skilled manual/non-manual | 43.0 | 42.0 |  |
| Semi-skilled/unskilled | 6.7 | 6.9 |  |
| Homemakers | 20.4 | 20.6 |  |
| Others | 2.1 | 2.7 |  |
| Region of nationality |  |  | 0.2 |
| Spain | 89.4 | 89.4 |  |
| Europe | 3.5 | 3.5 |  |
| Central and South America | 3.6 | 3.6 |  |
| Africa | 2.9 | 2.9 |  |
| Asia | 0.6 | 0.6 |  |
| Civil status (married vs. no-married) | 70.1 | 70.1 | 5.7 |
| **Paternal characteristics** |  |  |  |
| Age (years) | 34.2 (5.6) | 34.1 (5.7) | 1.5 |
| Educational level |  |  | 30.8 |
| High | 26.5 | 23.5 |  |
| Medium | 59.9 | 61.8 |  |
| Low | 13.5 | 14.7 |  |
| Social class based on occupation |  |  | 11.9 |
| Managers | 5.3 | 5.8 |  |
| Technicians | 24.2 | 23.9 |  |
| Skilled manual/non-manual | 40.0 | 39.4 |  |
| Semi-skilled/unskilled | 27.0 | 26.2 |  |
| Others | 3.5 | 4.7 |  |

Values are percentages for the categorical variables and mean (standard deviation) for the continuous variables.

There were no missing data on child sex, maternal age, parity, area-level deprivation index, urbanicity, mean temperature across pregnancy.

# **Table S3. Characteristics of the participants included and not included in the PM_10_ analysis**

|  | **Participants**  **(n=3,678,445)** | **Non-participants**  **(n=1,815,527)** | **p-value** |
| --- | --- | --- | --- |
| **Maternal characteristics** |  |  |  |
| Age (years) | 31.8 (5.2) | 30.8 (5.7) | <0.001 |
| *Missing (N (%))* | *0* | *0* |  |
| Educational level |  |  | <0.001 |
| High | 37.2 | 26.7 |  |
| Medium | 51.7 | 51.2 |  |
| Low | 11.0 | 22.0 |  |
| *Missing (N (%))* | *1,091,914 (29.7%)* | *582,701 (32.1%)* |  |
| Social class based on occupation |  |  | <0.001 |
| Managers | 3.1 | 3.0 |  |
| Technicians | 24.8 | 18.2 |  |
| Skilled manual/non-manual | 43.0 | 37.5 |  |
| Semi-skilled/unskilled | 6.7 | 8.6 |  |
| Homemakers | 20.4 | 30.2 |  |
| Others | 2.1 | 2.7 |  |
| *Missing (N (%))* | *364,314 (9.9%)* | *301,260 (16.6%)* |  |
| Region of nationality |  |  | <0.001 |
| Spain | 89.4 | 66.2 |  |
| Europe | 3.5 | 8.7 |  |
| Central and South America | 3.6 | 10.7 |  |
| Africa | 2.9 | 11.5 |  |
| Asia | 0.6 | 2.9 |  |
| *Missing (N (%))* | *7,879 (0.2%)* | *10,510 (0.6%)* |  |
| Civil status (married vs. no-married) | 70.1 | 67.1 | <0.001 |
| *Missing (N (%))* | *208,574 (5.7%)* | *127,886 (7.0%)* |  |
| Parity |  |  | <0.001 |
| 0 | 54.9 | 53.1 |  |
| 1 | 36.5 | 34.8 |  |
| 2 | 6.8 | 8.7 |  |
| 3 or more | 1.9 | 3.5 |  |
| *Missing (N (%))* | *0* | *0* |  |
| **Paternal characteristics** |  |  |  |
| Age (years) | 34.2 (5.6) | 34.1 (6.3) | <0.001 |
| *Missing (N (%))* | *56,070 (1.5%)* | *45,128 (2.5%)* |  |
| Educational level |  |  | <0.001 |
| High | 26.5 | 19.8 |  |
| Medium | 59.9 | 57.2 |  |
| Low | 13.5 | 23.0 |  |
| *Missing (N (%))* | *1,133,297 (30.8%)* | *610,676 (33.6%)* |  |
| Social class based on occupation |  |  | <0.001 |
| Managers | 5.3 | 5.3 |  |
| Technicians | 24.2 | 18.8 |  |
| Skilled manual/non-manual | 40.0 | 42.5 |  |
| Semi-skilled/unskilled | 27.0 | 29.4 |  |
| Others | 3.5 | 4.0 |  |
| *Missing (N (%))* | *439,199 (11.9%)* | *356,412 (19.6%)* |  |
| Values are percentages for the categorical variables and mean (standard deviation) for the continuous variables. P-values are based on chi-square tests for categorical variables and two-sample t-test for continuous variables. | | | |

**Table S3. Continuation**

|  | **Participants**  **(n=3,678,445)** | **Non-participants**  **(n=1,815,527)** | **p-value** |
| --- | --- | --- | --- |
| **Household characteristics** |  |  |  |
| Area-level deprivation index | -0.21 (0.99) | -0.02 (1.04) | <0.001 |
| *Missing (N (%))* | *0* | *1,066,142 (58.7%)* |  |
| Urbanicity |  |  | <0.001 |
| Cities | 55.9 | 51.9 |  |
| Towns or suburbs | 33.9 | 33.1 |  |
| Rural areas | 10.2 | 15.1 |  |
| *Missing (N (%))* | *0* | *1,066,142 (58.7%)* |  |
| Values are percentages for the categorical variables and mean (standard deviation) for the continuous variables. P-values are based on chi-square tests for categorical variables and two-sample t-test for continuous variables. | | | |

# **Table S4. Characteristics of the participants included and not included in the PM_2.5_ analysis**

|  | **Participants**  **(n=1,992,031)** | **Non-participants**  **(n=3,501,941)** | **p-value** |
| --- | --- | --- | --- |
| **Maternal characteristics** |  |  |  |
| Age (years) | 32.2 (5.2) | 31.0 (5.4) | <0.001 |
| *Missing (N (%))* | *0* | *0* |  |
| Educational level |  |  | <0.001 |
| High | 38.6 | 29.5 |  |
| Medium | 50.2 | 52.8 |  |
| Low | 11.1 | 17.7 |  |
| *Missing (N (%))* | *180,820 (9.1%)* | *1,493,795(42.6%)* |  |
| Social class based on occupation |  |  | <0.001 |
| Managers | 3.4 | 2.8 |  |
| Technicians | 26.9 | 20.1 |  |
| Skilled manual/non-manual | 42.2 | 40.7 |  |
| Semi-skilled/unskilled | 6.8 | 7.6 |  |
| Homemakers | 18.3 | 26.6 |  |
| Others | 2.4 | 2.2 |  |
| *Missing (N (%))* | *142,987 (7.2%)* | *522,587 (14.9%)* |  |
| Region of nationality |  |  | <0.001 |
| Spain | 87.8 | 78.3 |  |
| Europe | 4.3 | 5.8 |  |
| Central and South America | 3.6 | 7.3 |  |
| Africa | 3.5 | 7.0 |  |
| Asia | 0.7 | 1.7 |  |
| *Missing (N (%))* | *6,468 (0.3%)* | *11,921 (0.3%)* |  |
| Civil status (married vs. no-married) | 60.7 | 74.4 | <0.001 |
| *Missing (N (%))* | *0* | *336,460 (9.6%)* |  |
| Parity |  |  | <0.001 |
| 0 | 53.5 | 54.8 |  |
| 1 | 37.5 | 35.0 |  |
| 2 | 7.0 | 7.6 |  |
| 3 or more | 2.0 | 2.7 |  |
| *Missing (N (%))* | *0* | *0* |  |
| **Paternal characteristics** |  |  |  |
| Age (years) | 34.7 (5.6) | 34.1 (6.3) | <0.001 |
| *Missing (N (%))* | *34,749 (1.7%)* | *45,128 (2.5%)* |  |
| Educational level |  |  | <0.001 |
| High | 27.2 | 19.8 |  |
| Medium | 59.1 | 57.2 |  |
| Low | 13.6 | 23.0 |  |
| *Missing (N (%))* | *189,250 (9.5%)* | *610,676 (33.6%)* |  |
| Social class based on occupation |  |  | <0.001 |
| Managers | 5.4 | 5.3 |  |
| Technicians | 25.6 | 18.8 |  |
| Skilled manual/non-manual | 42.0 | 42.5 |  |
| Semi-skilled/unskilled | 22.7 | 29.4 |  |
| Others | 4.3 | 4.0 |  |
| *Missing (N (%))* | *189,510 (9.5%)* | *356,412 (19.6%)* |  |
| Values are percentages for the categorical variables and mean (standard deviation) for the continuous variables. P-values are based on chi-square tests for categorical variables and two-sample t-test for continuous variables. | | | |

**Table S4. Continuation**

|  | **Participants**  **(n=3,678,445)** | **Non-participants**  **(n=1,815,527)** | **p-value** |
| --- | --- | --- | --- |
| **Household characteristics** |  |  |  |
| Area-level deprivation index | -0.23 (0.98) | -0.02 (1.04) | <0.001 |
| *Missing (N (%))* | *0* | *1,066,142 (58.7%)* |  |
| Urbanicity |  |  | <0.001 |
| Cities | 55.5 | 51.9 |  |
| Towns or suburbs | 34.1 | 33.1 |  |
| Rural areas | 10.4 | 15.1 |  |
| *Missing (N (%))* | *0* | *1,066,142 (58.7%)* |  |
| Values are percentages for the categorical variables and mean (standard deviation) for the continuous variables. P-values are based on chi-square tests for categorical variables and two-sample t-test for continuous variables. | | | |

# **Table S5. List of variables explored to calculate the inverse probability of attrition weights and those included in the analysis**

| **Variables explored** | **Variables included** |
| --- | --- |
| **Maternal characteristics** |  |
| Age | X |
| Education level | X |
| Social class | X |
| Region of nationality | X |
| Civil status | X |
| Parity | X |
| **Paternal characteristics** |  |
| Age | X |
| Education level |  |
| Social class | X |
| **Household characteristics** |  |
| Area-level deprivation level | X |
| Urbanicity | X |
| Mean temperature across pregnancy |  |
| **Seasonality** |  |
| Season of conception |  |

|  | **Birthweight** | **Birthweight at term** |
| --- | --- | --- |
| **PM_10_** | 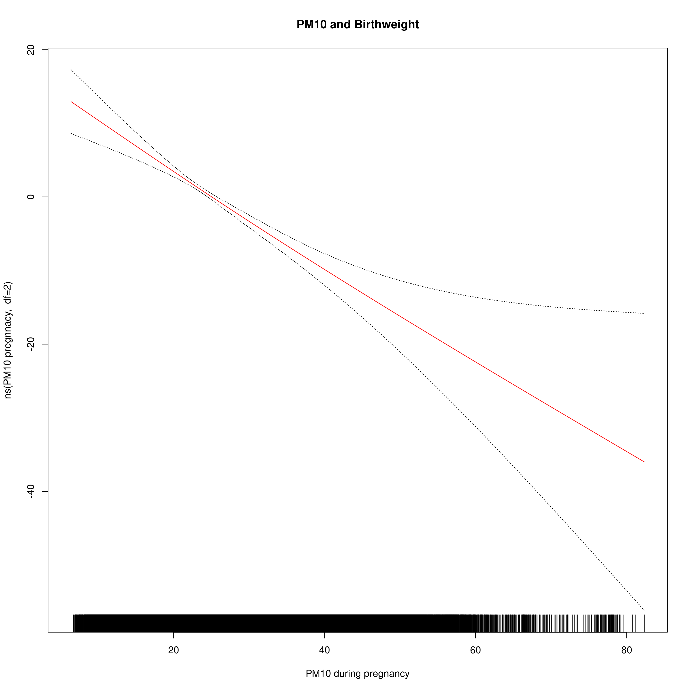 | 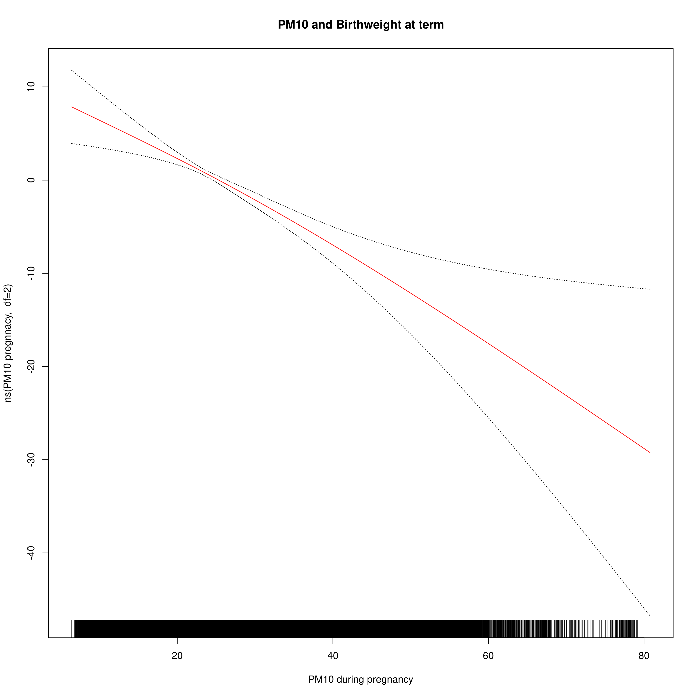 |
| **PM_2.5_** | 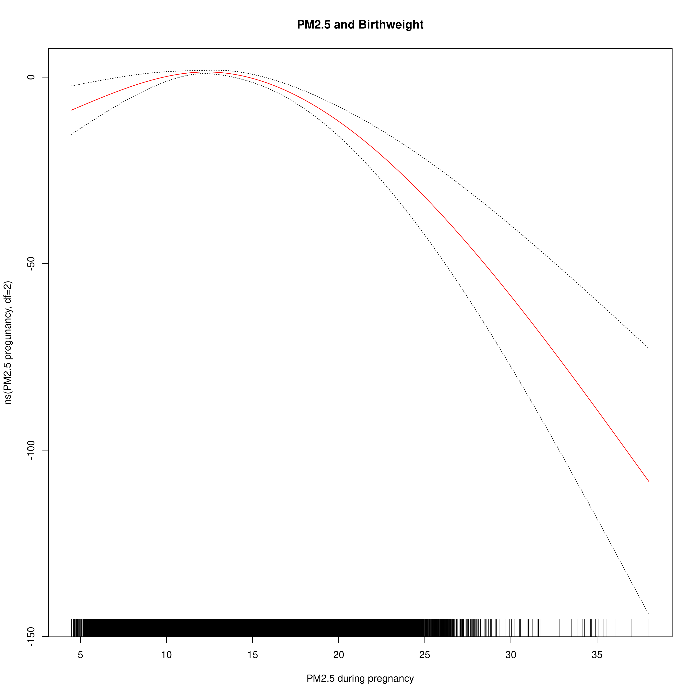 | 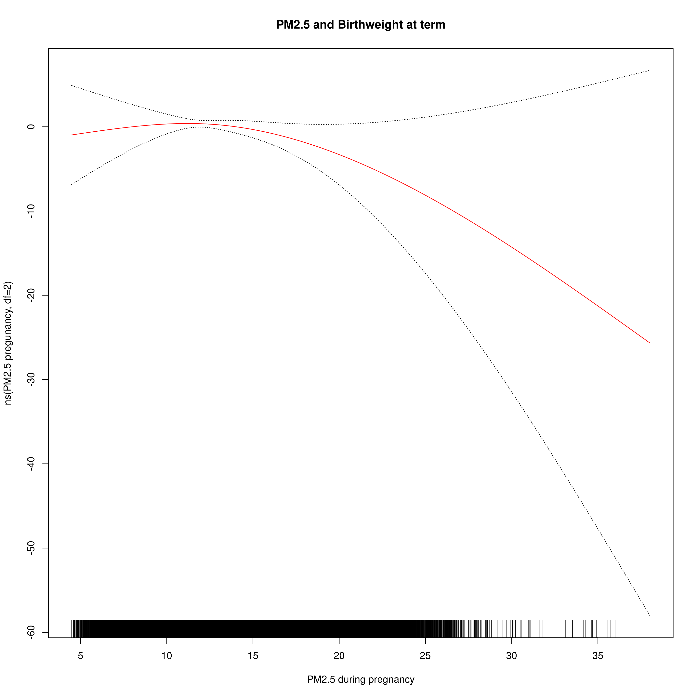 |

# **Figure S1. Shape of the association between pregnancy average particulate matter concentrations and birth outcomes**

Abbreviations: PM_10_, particular matter with aerodynamic diameter less than 10μm; PM_2.5_, particular matter with aerodynamic diameter less than 2.5μm.

Generalized additive models adjusted for parental age, parental educational level, parental social class based on occupation, maternal nationality, maternal civil status, parity, area-level deprivation index, urbanicity, month and year of conception, mean temperature across pregnancy, and geographical region.

|  | **Low birthweight at term** | **Preterm birth** |
| --- | --- | --- |
| **PM_10_** | 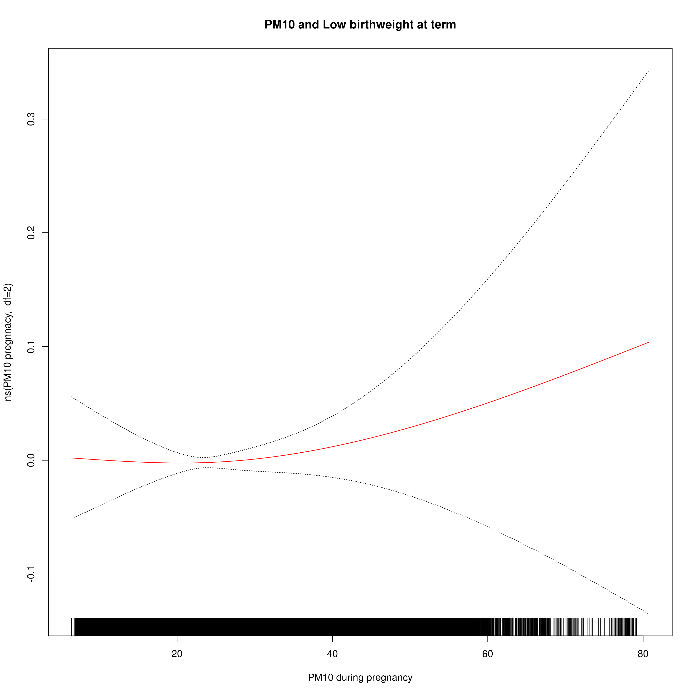 | 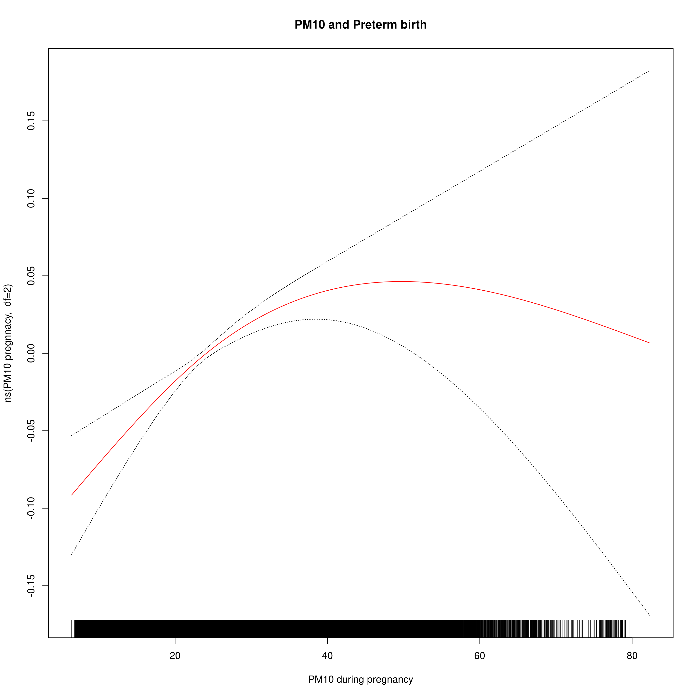 |
| **PM_2.5_** | 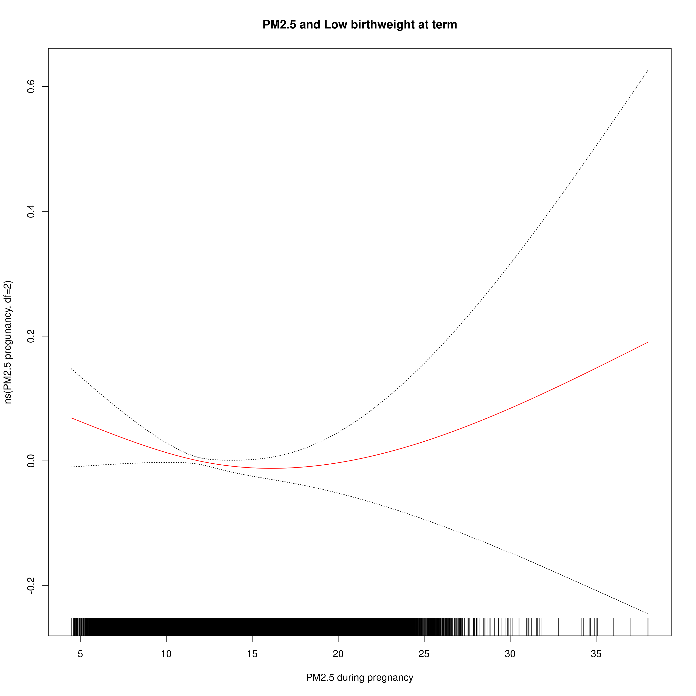 | 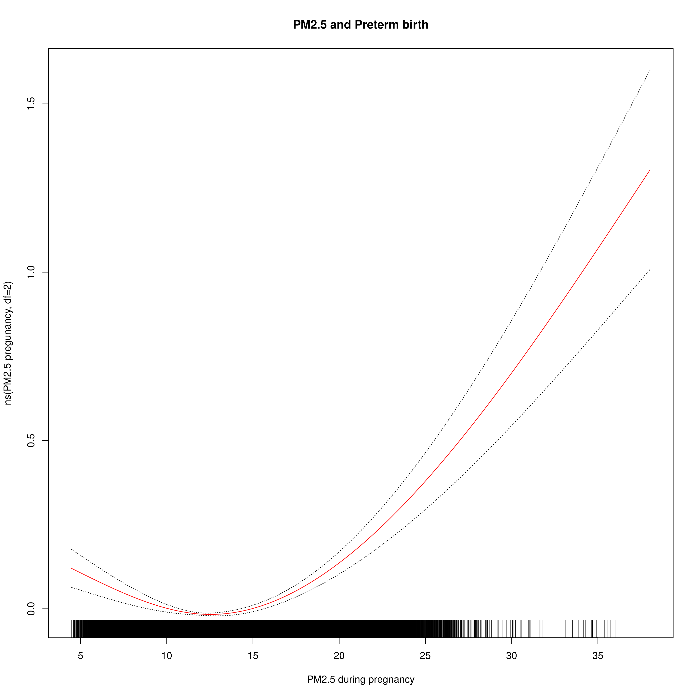 |

**Figure S1. Continuation**

|  | **Small for gestational age** |
| --- | --- |
| **PM_10_** | 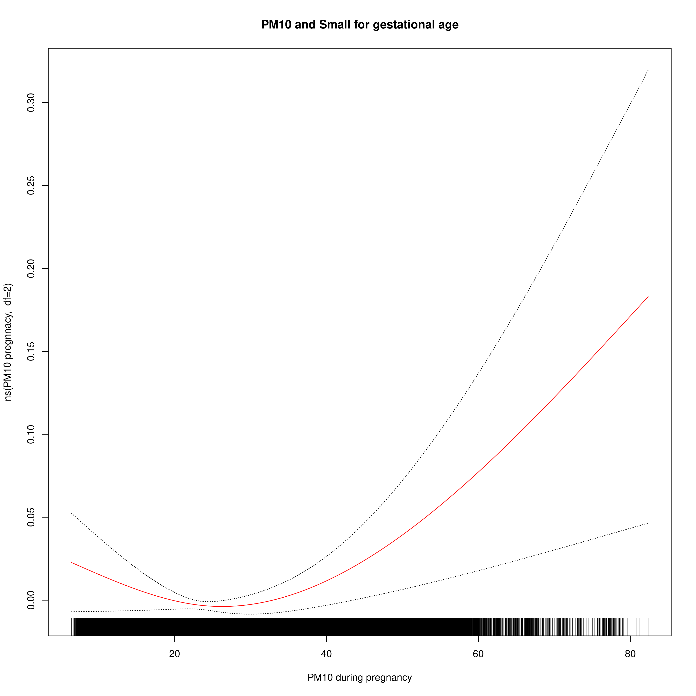 |
| **PM_2.5_** | 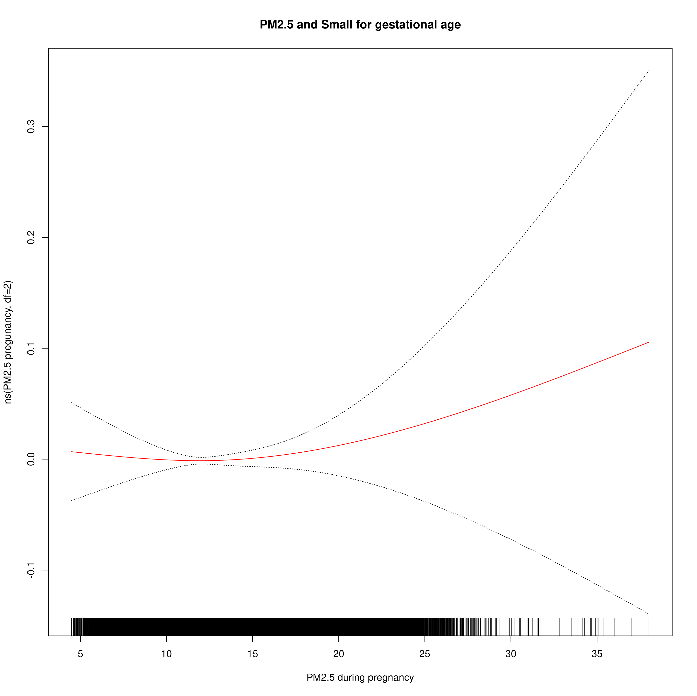 |

**Figure S1. Continuation**

| **Week 1** | **Week 5** |
| --- | --- |
| 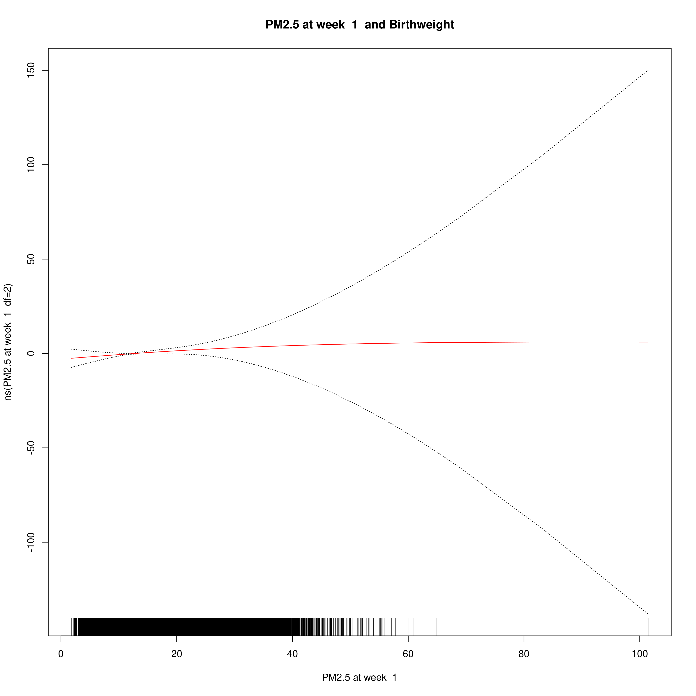 | 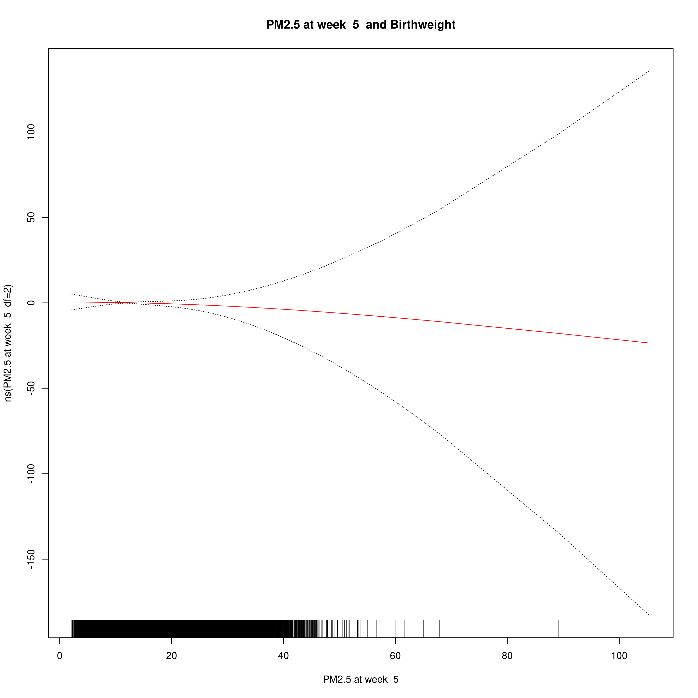 |
| **Week 10** | **Week 15** |
| 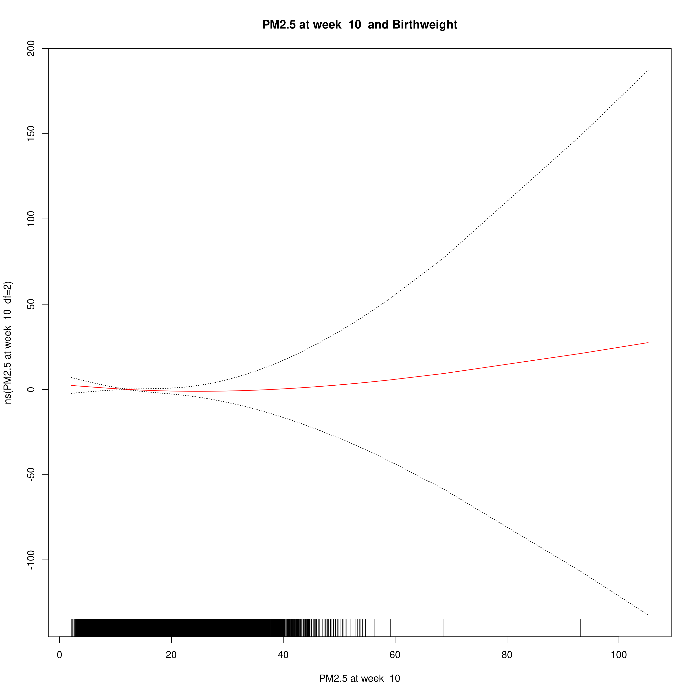 | 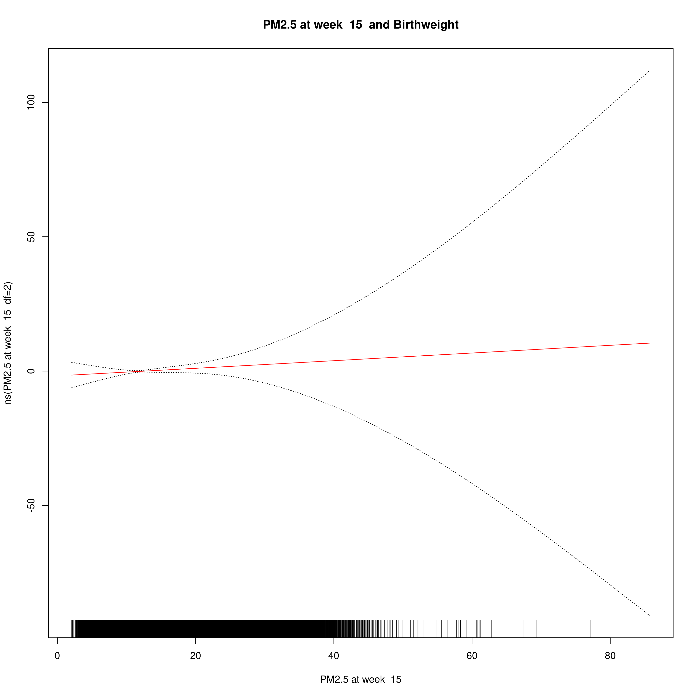 |

# **Figure S2. Shape of the association of PM_2.5_ exposure at weeks 1, 5, 10, 15, 20, 25, 30, and 32 with birthweight**

Abbreviations: PM_10_, particular matter with aerodynamic diameter less than 10μm; PM_2.5_, particular matter with aerodynamic diameter less than 2.5μm.

Generalized additive models adjusted for parental age, parental educational level, parental social class based on occupation, maternal nationality, maternal civil status, parity, area-level deprivation index, urbanicity, month and year of conception, mean temperature across pregnancy, and autonomous community.

| **Week 20** | **Week 25** |
| --- | --- |
| 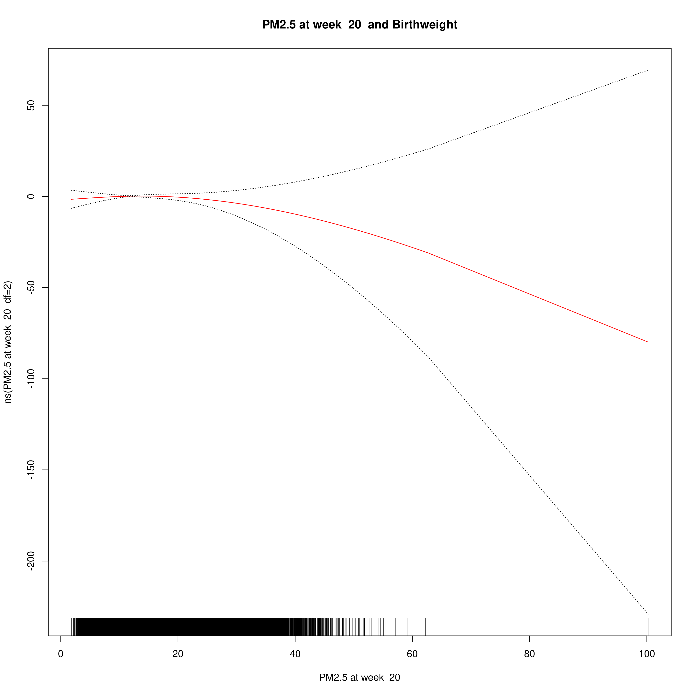 | 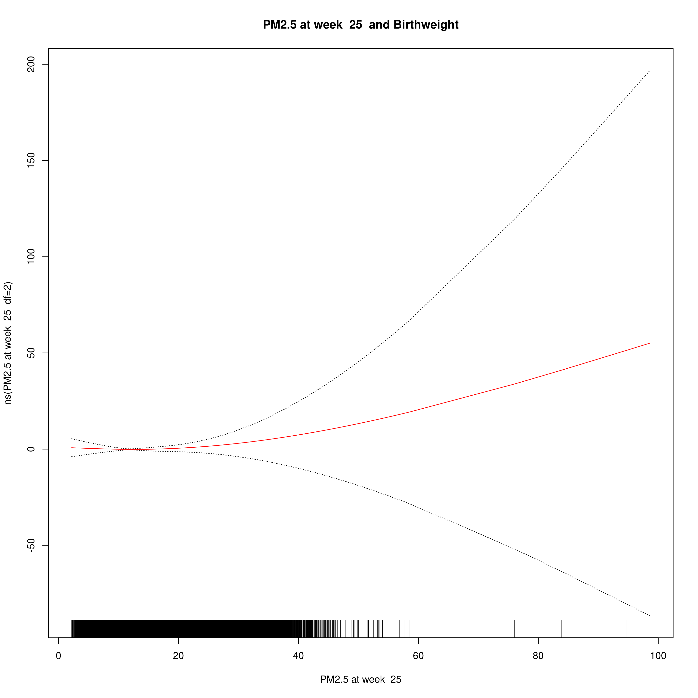 |
| **Week 30** | **Week 32** |
| 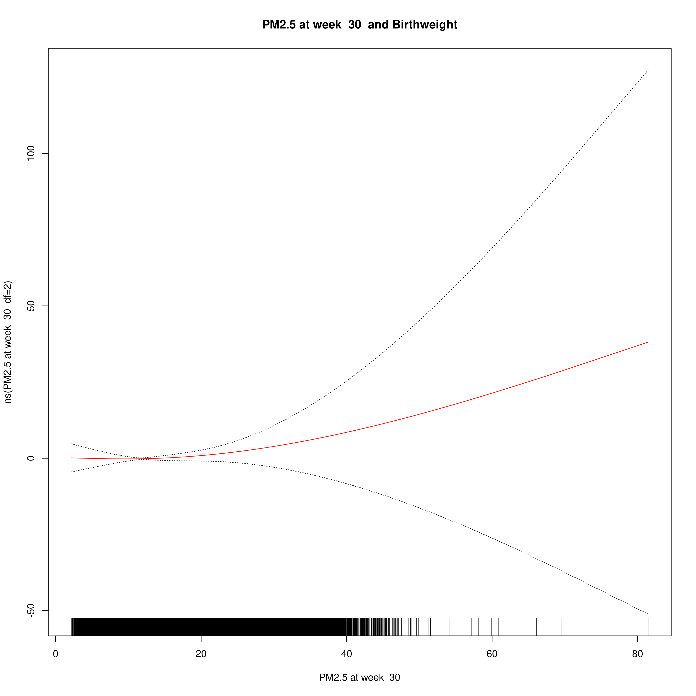 | 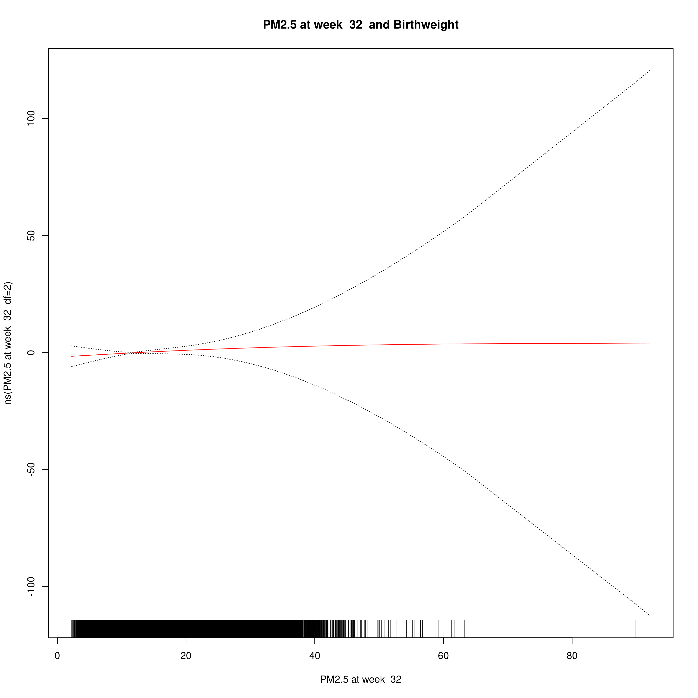 |

**Figure S2. Continuation**

# **Methods S2. Equation of the distributed lag model**

$$g(Y_{t}) \sim\beta_{0}+ \sum_{l=0}^{L} f\left( l \right){\cdot x}_{t-l}+\sum_{l=0}^{L} h\cdot z({temp}_{t-l},l)+ \gamma X+ \epsilon$$

where:

- $Y:$ outcome (here: birthweight or small for gestational age)
- $g:$ link function (here: identity for birthweight, logit for small for gestational age)
- $\beta_{0}:\mathrm{intercept}$
- $l$: lag number, ranging from 0 to $L$ (here: $L=31$), where each lag corresponds to one week of gestational age, with $l=0$ representing the first week of gestation
- $f\left( l \right)\cdot x_{t-l}$: lag-response function for air pollutants (${PM}_{10}or {PM}_{2.5}$); The exposure-response function is linear
- $h\cdot z({temp}_{t-l},l)$: exposure-lag-response function for temperature; Temperature is included using a natural cubic spline in both the exposure and lag dimension, with internal knots placed at the 25^th^ and 75^th^ percentiles of the temperature distribution and a knot at lag 50^th^ percentile for the lag-response function
- $X: vector of covariates$ – independent covariates, including parental age, parental educational level, parental social class based on occupation, maternal nationality, maternal civil status, parity, area-level deprivation index, urbanicity, month and year of conception, and geographical region
- $\epsilon:error term$
- $t:$ index for individual births (one observation per participant)

# **Table S6. Distribution of particle matter concentrations during pregnancy**

|  | **Mean** | **SD** | **Min** | **P25** | **P50** | **P75** | **Max** |
| --- | --- | --- | --- | --- | --- | --- | --- |
| **PM_10_ (µg/m^3^)** | 25.1 | 6.6 | 6.4 | 20.1 | 24.2 | 29.9 | 82.4 |
| **PM_2.5_ (µg/m^3^)** | 12.7 | 2.4 | 4.5 | 11.0 | 12.3 | 14.1 | 38.0 |

Abbreviation: SD, standard deviation; Min, minimum; Max, maximum; P, percentile; PM_10_, particular matter with aerodynamic diameter less than 10μm; PM_2.5_, particular matter with aerodynamic diameter less than 2.5μm


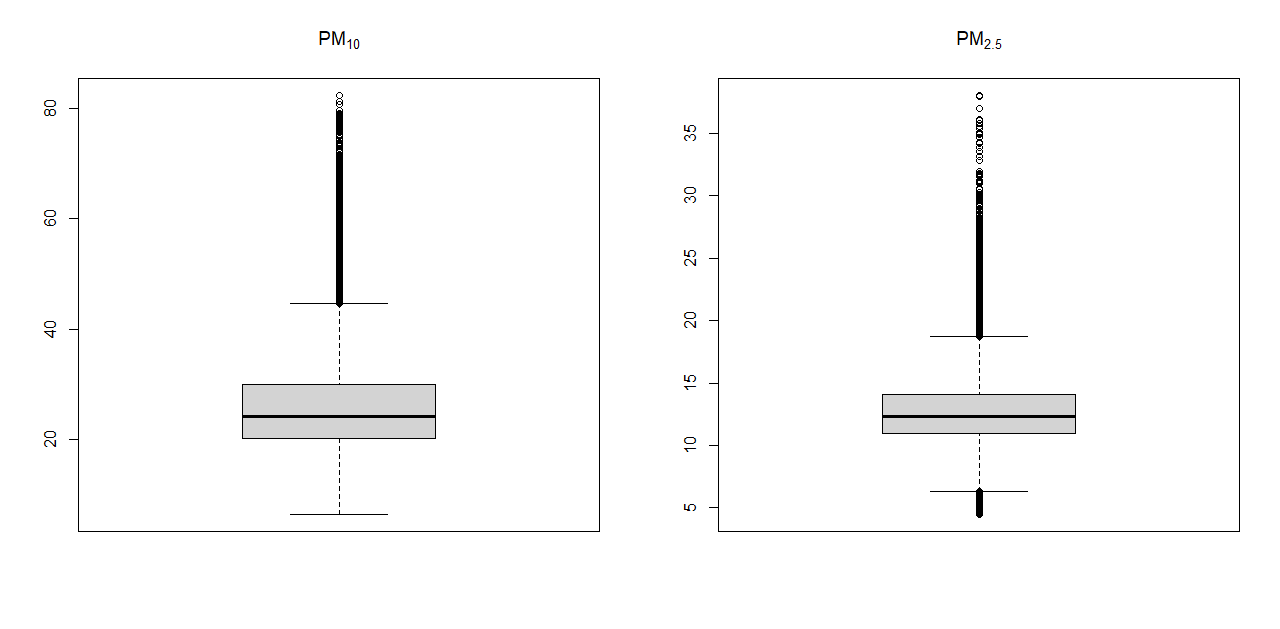


# **Figure S3. Distribution of particle matter concentrations during pregnancy**

PM_10_, particular matter with aerodynamic diameter less than 10μm; PM_2.5_, particular matter with aerodynamic diameter less than 2.5μm

# **Table S7. Characteristics of the participants with pregnancy-average PM_2.5_ levels below and above** **10 μg/m^3^**

|  | **PM_2.5_ ≤ 10 μg/m^3^ (n=220,352)** | **PM_2.5_ > 10 μg/m^3^ (n=1,771,679)** | **p-value** |
| --- | --- | --- | --- |
| **Maternal characteristics** |  |  |  |
| Age (years) | 32.7 (5.2) | 32.2 (5.7) | <0.001 |
| Educational level |  |  | <0.001 |
| High | 39.4 | 38.6 |  |
| Medium | 51.0 | 50.1 |  |
| Low | 9.6 | 11.3 |  |
| Social class based on occupation |  |  | <0.001 |
| Managers | 3.2 | 3.5 |  |
| Technicians | 28.4 | 26.7 |  |
| Skilled manual/non-manual | 40.6 | 42.3 |  |
| Semi-skilled/unskilled | 7.7 | 6.7 |  |
| Homemakers | 17.9 | 18.3 |  |
| Others | 2.2 | 2.4 |  |
| Region of nationality |  |  | <0.001 |
| Spain | 89.0 | 87.7 |  |
| Europe | 4.7 | 4.3 |  |
| Central and South America | 2.6 | 3.7 |  |
| Africa | 3.2 | 3.6 |  |
| Asia | 0.5 | 0.8 |  |
| Civil status (married vs. no-married) | 59.9 | 60.7 | <0.001 |
| Parity |  |  | 0.003 |
| 0 | 53.4 | 53.5 |  |
| 1 | 37.6 | 37.5 |  |
| 2 | 7.1 | 7.0 |  |
| 3 or more | 1.9 | 2.0 |  |
| **Paternal characteristics** |  |  |  |
| Age (years) | 35.3 (5.6) | 34.6 (6.3) | <0.001 |
| Educational level |  |  | <0.001 |
| High | 24.5 | 27.6 |  |
| Medium | 62.7 | 58.7 |  |
| Low | 12.7 | 13.7 |  |
| Social class based on occupation |  |  | <0.001 |
| Managers | 4.9 | 5.5 |  |
| Technicians | 23.9 | 25.8 |  |
| Skilled manual/non-manual | 41.9 | 42.0 |  |
| Semi-skilled/unskilled | 25.0 | 22.4 |  |
| Others | 4.2 | 4.3 |  |
| **Household characteristics** |  |  |  |
| Area-level deprivation index | -0.20 (0.97) | -0.23 (0.98) | <0.001 |
| Urbanicity |  |  | <0.001 |
| Cities | 31.2 | 58.5 |  |
| Towns or suburbs | 31.2 | 34.2 |  |
| Rural areas | 37.5 | 7.1 |  |
| Temperature during pregnancy | 14.9 (3.0) | 16.6 (2.9) | <0.001 |
| Values are percentages for the categorical variables and mean (standard deviation) for the continuous variables. P-values are based on chi-square tests for categorical variables and two-sample t-test for continuous variables. | | | |

# **Table S8. Distribution of birth outcomes**

|  | **N** | **Distribution** | |
| --- | --- | --- | --- |
| **Birthweight (grams)** | 3,678,445 | 3248 | (495) |
| **Birthweight at term (grams)** | 3,468,035 | 3298 | (436) |
| **Low birthweight at term (yes *vs.* no)** | 3,468,035 | 2.9 |  |
| **Preterm birth (yes *vs.* no)** | 3,678,445 | 5.7 |  |
| **Small for gestational age (yes *vs.* no)** | 3,678,445 | 9.7 |  |

Distribution values are percentages for the categorical variables and mean (standard deviation) for the continuous variables.

# **Table S9. Spearman correlation between birth outcomes**

|  | **Low birthweight at term** | **Preterm birth** | **Small for gestational age** |
| --- | --- | --- | --- |
| **Birthweight** | -0.40 | -0.31 | -0.46 |
| **Low birthweight at term** | --- | 0.48 | 0.37 |
| **Preterm birth** | --- | --- | 0.00 |

# **Table S10. Adjusted association of pregnancy-average PM_2.5_ concentrations with birthweight and preterm birth below and above 10 μg/m^3^, overall and according to maternal educational level and to area-level deprivation index**

|  | **Birthweight** | | **Preterm birth** | |
| --- | --- | --- | --- | --- |
|  | **B** | **(95% CI)** | **OR** | **(95% CI)** |
| **PM_2.5_** **≤ 10 μg/m^3^ (∆ 5 μg/m^3^)** | (N=220,352) | | (N=220,352) | |
| Overall association | 1.9 | (-12.9, 16.7) | 0.90 | (0.79, 1.03) |
| By maternal educational level |  |  |  |  |
| High | 20.7 | ( -3.4, 44.1) | 0.95 | (0.75, 1.20) |
| Medium | -6.4 | (-26.9, 14.1) | 0.89 | (0.74, 1.06) |
| Low | 19.2 | (-67.5, 29.1) | 0.91 | (0.63, 1.32) |
| By area-level deprivation index |  |  |  |  |
| Low | 24.8 | ( -4.9, 54.5) | 0.93 | (0.70, 1.22) |
| Moderate | -10.6 | (-36.7, 15.5) | 0.86 | (0.68, 1.09) |
| High | 1.2 | (-22.2, 24.5) | 0.91 | (0.74, 1.11) |
| By maternal educational level &  area-level deprivation index |  |  |  |  |
| High education / Low deprivation | **40.9** | **( 0.4, 81.4)** | 1.02 | (0.68, 1.54) |
| High education / High deprivation | 3.3 | ( -40.5, 47.2) | 0.81 | (0.53, 1.23) |
| Low education / Low deprivation | 31.3 | (-104.7, 167.3) | 0.81 | (0.28, 2.32) |
| Low education / High deprivation | -35.2 | ( -99.2, 28.8) | 1.16 | (0.70, 1.91) |

Abbreviation: B, Beta coefficient, CI, Confidence interval, OR, Odds ratio, PM_2.5_, particular matter with aerodynamic diameter less than 2.5μm

Adjusted for parental age, parental educational level, parental social class based on occupation, maternal nationality, maternal civil status, parity, area-level deprivation index, urbanicity, month and year of conception, mean temperature across pregnancy, and geographical region. Stratified models were not adjusted for the stratification variable (i.e., maternal educational level and/or area-level deprivation index).

In bold, associations with a p-value ≤ 0.05. *Associations that survive correction for multiple testing (p-value ≤ 0.017)

**Table S10**. **Continuation**

|  | **Birthweight** | | **Preterm birth** | |
| --- | --- | --- | --- | --- |
|  | **B** | **(95% CI)** | **OR** | **(95% CI)** |
| **PM_2.5_ >** **10 μg/m^3^ (∆ 5 μg/m^3^)** | (N=1,771,679) | | (N=1,771,679) | |
| Overall association | **-5.4** | **( -7.7, -3.1)*** | **1.06** | **(1.04, 1.09)*** |
| By maternal educational level |  |  |  |  |
| High | **-5.0** | **( -8.2, -1.2)*** | **1.07** | **(1.03, 1.10)*** |
| Medium | **-3.7** | **( -7.0, -0.5)** | **1.06** | **(1.03, 1.09)*** |
| Low | **-14.5** | **(-21.8, -7.3)*** | **1.09** | **(1.03, 1.15)*** |
| By area-level deprivation index |  |  |  |  |
| Low | -2.7 | ( -6.5, 1.0) | 1.02 | (0.99, 1.07) |
| Moderate | **-7.9** | **(-11.8, -3.9)*** | **1.09** | **(1.05, 1.13)*** |
| High | **-7.6** | **(-11.8, -3.3)*** | **1.09** | **(1.05, 1.12)*** |
| By maternal educational level &  area-level deprivation index |  |  |  |  |
| High education / Low deprivation | **-5.3** | **(-10.2, - 0.5)** | **1.05** | **(1.00, 1.10)** |
| High education / High deprivation | -4.7 | (-13.2, 3.8) | **1.12** | **(1.04, 1.22)*** |
| Low education / Low deprivation | -2.1 | (-20.9, 16.7) | 1.05 | (0.91, 1.21) |
| Low education / High deprivation | **-14.4** | **( 24.4, -4.4)*** | **1.11** | **(1.03, 1.19)*** |

# **Table S11. Adjusted association of pregnancy-average PM_10_ and PM_2.5_ concentrations with birthweight after applying indirect adjustment for smoking use during pregnancy and pre-pregnancy body mass index**

|  | **Birthweight** | |
| --- | --- | --- |
|  | **B** | **(95% CI)** |
| **PM_10_ (∆ 10 μg/m^3^)** |  |  |
| Original association | -7.1 | ( -8.5, -5.7) |
| Indirect adjustment for smoking use during pregnancy | -11.3 | (-21.3, -1.2) |
| Indirect adjustment for pre-pregnancy body mass index | -7.0 | ( -8.4, -5.6) |
| Indirect adjustment for smoking use during pregnancy and pre-pregnancy body mass index | -11.1 | (-21.1, -1.1) |
| **PM_2.5_ (∆ 5 μg/m^3^)** |  |  |
| Original association | -3.8 | ( -5.9, -1.7) |
| Indirect adjustment for smoking use during pregnancy | -9.2 | (-22.9, 4.4) |
| Indirect adjustment for pre-pregnancy body mass index | -3.8 | ( -5.9, -1.6) |
| Indirect adjustment for smoking use during pregnancy and pre-pregnancy body mass index | -9.2 | (-22.8, 4.5) |

Abbreviation: B, Beta coefficient, CI, Confidence interval, PM_10_, particular matter with aerodynamic diameter less than 10μm; PM_2.5_, particular matter with aerodynamic diameter less than 2.5μm

Adjusted for parental age, parental educational level, parental social class based on occupation, maternal nationality, maternal civil status, parity, area-level deprivation index, urbanicity, month and year of conception, mean temperature across pregnancy, and geographical region.

# **Table S12. Characteristics of the analytical and the ancillary cohort**

|  | **Distribution**  **(Percentage or Mean (SD))** | |
| --- | --- | --- |
|  | **Analytical cohort**  **(N=3,678,445)** | **Ancillary cohort**  **(N=2,487)** |
| **Maternal characteristics** |  |  |
| Age (years) | 31.8 (5.2) | 31.9 (4.3) |
| Educational level |  |  |
| High | 33.0 | 34.3 |
| Medium | 54.7 | 41.3 |
| Low | 12.3 | 24.4 |
| Social class based on occupation |  |  |
| Managers/ Technicians | 27.8 | 21.5 |
| Skilled manual/non-manual | 42.0 | 25.9 |
| Semi-skilled/unskilled | 6.9 | 52.6 |
| Homemakers | 20.6 | 0.0 |
| Others | 2.7 | 0.0 |
| Region of nationality |  |  |
| Spain | 89.4 | 91.9 |
| Other | 10.6 | 8.1 |
| Civil status (married vs. no-married) | 70.1 | 98.3 |
| Parity |  |  |
| No children | 54.9 | 56.3 |
| 1 child | 36.5 | 36.9 |
| 2 or more children | 8.7 | 6.8 |
| **Paternal characteristics** |  |  |
| Age (years) | 34.1 (5.7) | 34.0 (5.0) |
| Educational level |  |  |
| High | 23.5 | 20.7 |
| Medium | 61.8 | 43.8 |
| Low | 14.7 | 35.5 |
| Social class based on occupation |  |  |
| Managers/ Technicians | 29.7 | 19.7 |
| Skilled manual/non-manual | 39.4 | 17.2 |
| Semi-skilled/unskilled | 26.2 | 63.0 |
| Others | 4.7 | 0.1 |
| **Household characteristics** |  |  |
| Area-level deprivation index | -0.21 (0.99) | -0.30 (0.70) |
| Urbanicity |  |  |
| Cities | 55.9 | 81.5 |
| Towns or suburbs | 33.9 | 14.2 |
| Rural areas | 10.2 | 4.3 |
| Mean temperature across pregnancy | 16.2 (2.9) | 16.2 (2.4) |
| **Particle matter concentrations (**µg/m^3^) |  |  |
| PM_10_ | 25.1 (6.6) | 31.2 (6.2) |
| PM_2.5_ | 12.7 (2.4) | 14.8 (3.3) |

Abbreviations: PM_10_, particular matter with aerodynamic diameter less than 10μm; PM_2.5_, particular matter with aerodynamic diameter less than 2.5μm

# **Table S13. Adjusted lag-response association of weekly-average PM_10_ and PM_2.5_ concentrations with birthweight and small for gestational age according to maternal educational level and area-level deprivation index**

|  | **Birthweight** | | | **Small for gestational age** | | |
| --- | --- | --- | --- | --- | --- | --- |
|  | **Lags** | **B** | **(95% CI)** | **Lags** | **OR** | **(95% CI)** |
| **PM_10_ (∆ 10 μg/m^3^)** |  |  | |  |  | |
| Overall | Weeks 1-4 | -1.3 | ( -2.6, -0.3) | --- | --- | --- |
|  | Weeks 12-13 | 0.3 | ( 0.0, 0.6) |  |  |  |
|  | Weeks 23-32 | -3.6 | ( -4.9, -2.2) | --- | --- | --- |
| **PM_2.5_ (∆ 5 μg/m^3^)** |  |  | |  |  | |
| Overall | --- | --- | --- | --- | --- | --- |

Abbreviation: B, Beta coefficient, CI, Confidence interval, OR, Odds ratio, PM_2.5_, particular matter with aerodynamic diameter less than 2.5μm

Adjusted for parental age, parental educational level, parental social class based on occupation, maternal nationality, maternal civil status, parity, area-level deprivation index, urbanicity, month and year of conception, weekly temperature across pregnancy, and geographical region


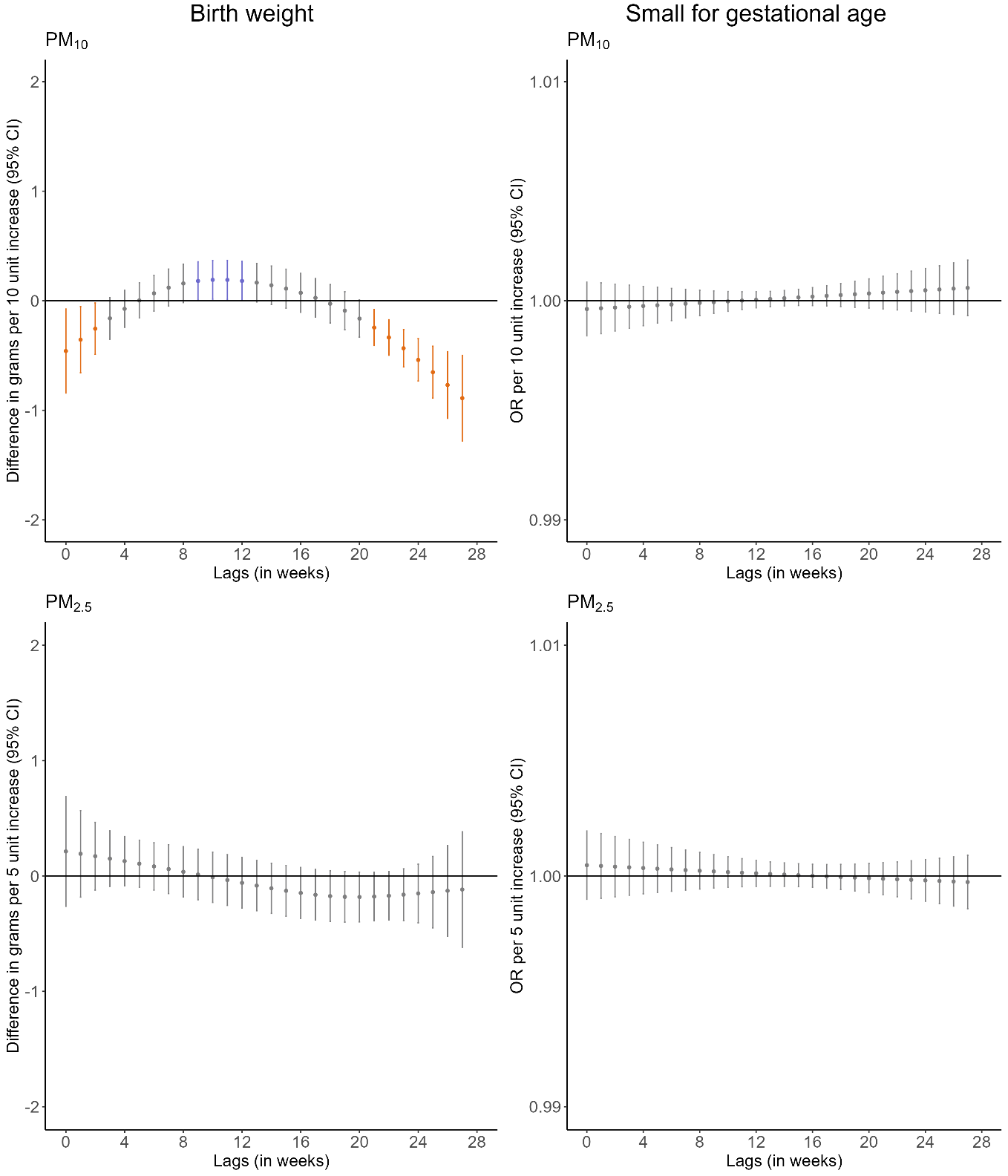


# **Figure S4. Adjusted lag-response association of weekly-average PM_10_ and PM_2.5_ concentrations during pregnancy with birthweight and small for gestational age including all participants born from week 28 onwards**

Abbreviations: CI, confidence interval; OR, odds ratio; PM_10_, particular matter with aerodynamic diameter less than 10μm; PM_2.5_, particular matter with aerodynamic diameter less than 2.5μm

Adjusted for parental age, parental educational level, parental social class based on occupation, maternal nationality, maternal civil status, parity, area-level deprivation index, month and year of conception, weekly temperature across pregnancy, and geographical region

Dots represent the effect estimates of the association between the exposure at each specific lag and the outcome. Vertical gray, blue, and orange lines represent 95% CI and indicate no divergence from the null, significant divergence from positive association, and significant divergence from negative association, respectively. All associations survived correction for multiple testing (p-value ≤ 0.05)


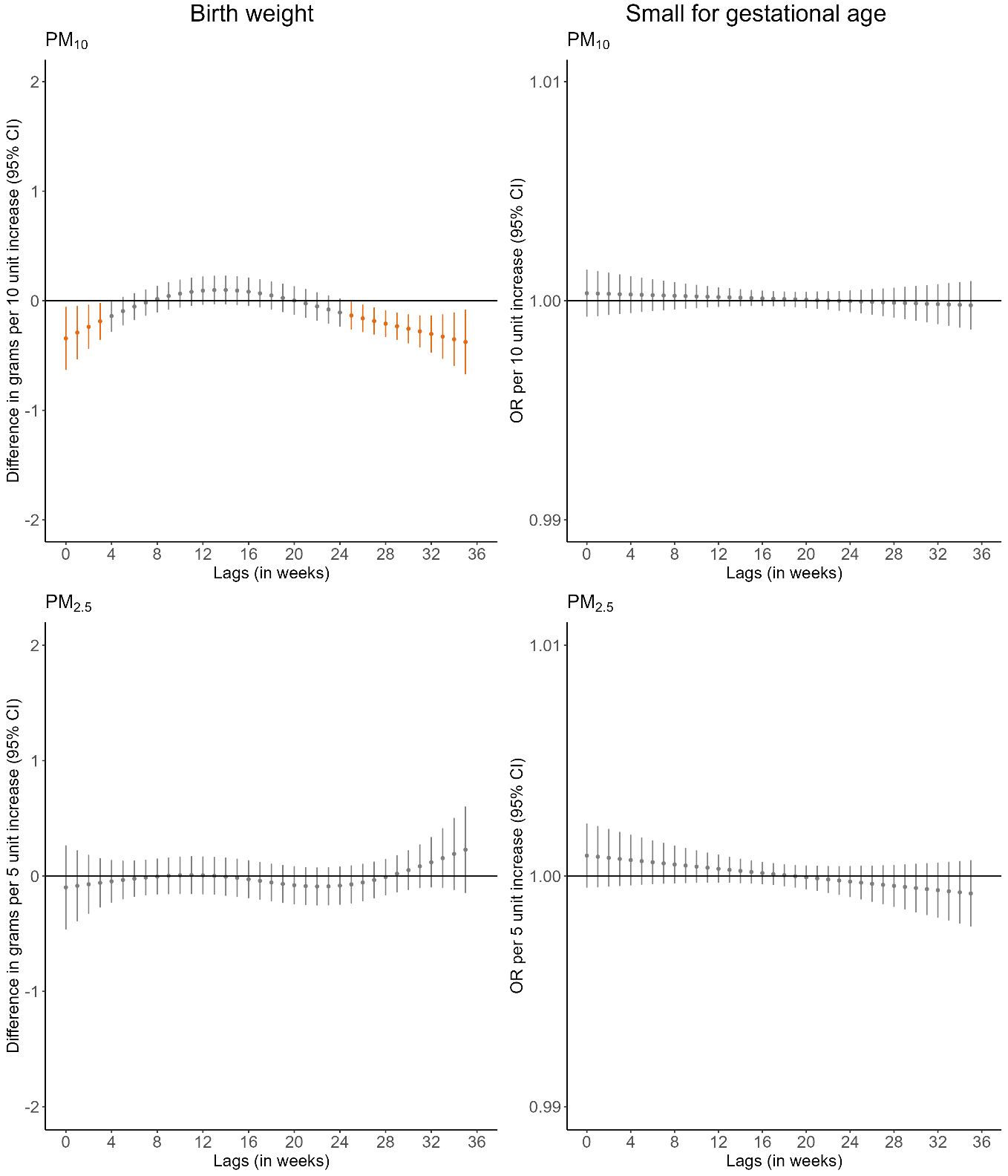


# **Figure S5. Adjusted lag-response association of weekly-average PM_10_ and PM_2.5_ concentrations during pregnancy with birthweight and small for gestational age including all participants born from week 36 onwards**

Abbreviations: CI, confidence interval; OR, odds ratio; PM_10_, particular matter with aerodynamic diameter less than 10μm; PM_2.5_, particular matter with aerodynamic diameter less than 2.5μm

Adjusted for parental age, parental educational level, parental social class based on occupation, maternal nationality, maternal civil status, parity, area-level deprivation index, month and year of conception, weekly temperature across pregnancy, and geographical region

Dots represent the effect estimates of the association between the exposure at each specific lag and the outcome. Vertical gray, blue, and orange lines represent 95% CI and indicate no divergence from the null, significant divergence from positive association, and significant divergence from negative association, respectively. All associations survived correction for multiple testing (p-value ≤ 0.05)

# **Table S14. Adjusted lag-response association of weekly-average PM_10_ and PM_2.5_ concentrations with birthweight and small for gestational age according to maternal educational level and area-level deprivation index**

|  | **Birthweight** | | | **Small for gestational age** | | |
| --- | --- | --- | --- | --- | --- | --- |
|  | **Lags** | **B** | **(95% CI)** | **Lags** | **OR** | **(95% CI)** |
| **PM_10_ (∆ 10 μg/m^3^)** |  |  | |  |  | |
| By maternal educational level^1^ |  |  |  |  |  |  |
| High | Weeks 27-30 | -1.3 | ( -2.4, -0.2) | --- | --- | --- |
| Medium | Weeks 0-3 | -1.3 | ( -2.4, -0.2) | --- | --- | --- |
|  | Weeks 10-16 | 1.8 | ( 0.5, 3.1) | --- | --- | --- |
|  | Weeks 24-32 | -4.0 | ( -5.9, -2.2) | --- | --- | --- |
| Low | Weeks 13-30 | -7.2 | (-10.8, -3.5) | --- | --- | --- |
| By area-level deprivation index^2^ |  |  |  |  |  |  |
| Low | Weeks 10-14 | 1.3 | ( 0.2, 2.4) | --- | --- | --- |
|  | Weeks 25-32 | -3.3 | ( -5.5, -1.2) | --- | --- | --- |
| Moderate | Weeks 1-4 | -1.9 | ( -3.6, -0.3) | --- | --- | --- |
|  | Weeks 24-31 | -2.9 | ( -4.8, -1.0) | --- | --- | --- |
| High | Weeks 24-31 | -2.9 | ( -4.8, -0.9) | --- | --- | --- |
| **PM_2.5_ (∆ 5 μg/m^3^)** |  |  | |  |  | |
| By maternal educational level^1^ |  |  |  |  |  |  |
| High | --- | --- | --- | --- | --- | --- |
| Medium | --- | --- | --- | --- | --- | --- |
| Low | Weeks 18-23 | -2.9 | ( -5.5, -0.3) | --- | --- | --- |
| By area-level deprivation index^2^ |  |  |  |  |  |  |
| Low | --- | --- | --- | --- | --- | --- |
| Moderate | --- | --- | --- | --- | --- | --- |
| High | Weeks 21-25 | -1.2 | ( -2.2, -0.1) | --- | --- | --- |

Abbreviation: B, Beta coefficient, CI, Confidence interval, OR=Odds ratio, PM_2.5_, particular matter with aerodynamic diameter less than 2.5μm

^1^Adjusted for parental age, paternal educational level, parental social class based on occupation, maternal nationality, maternal civil status, parity, area-level deprivation index, urbanicity, month and year of conception, weekly temperature across pregnancy, and geographical region

^2^Adjusted for parental age, parental educational level, parental social class based on occupation, maternal nationality, maternal civil status, parity, urbanicity, month and year of conception, weekly temperature across pregnancy, and geographical region


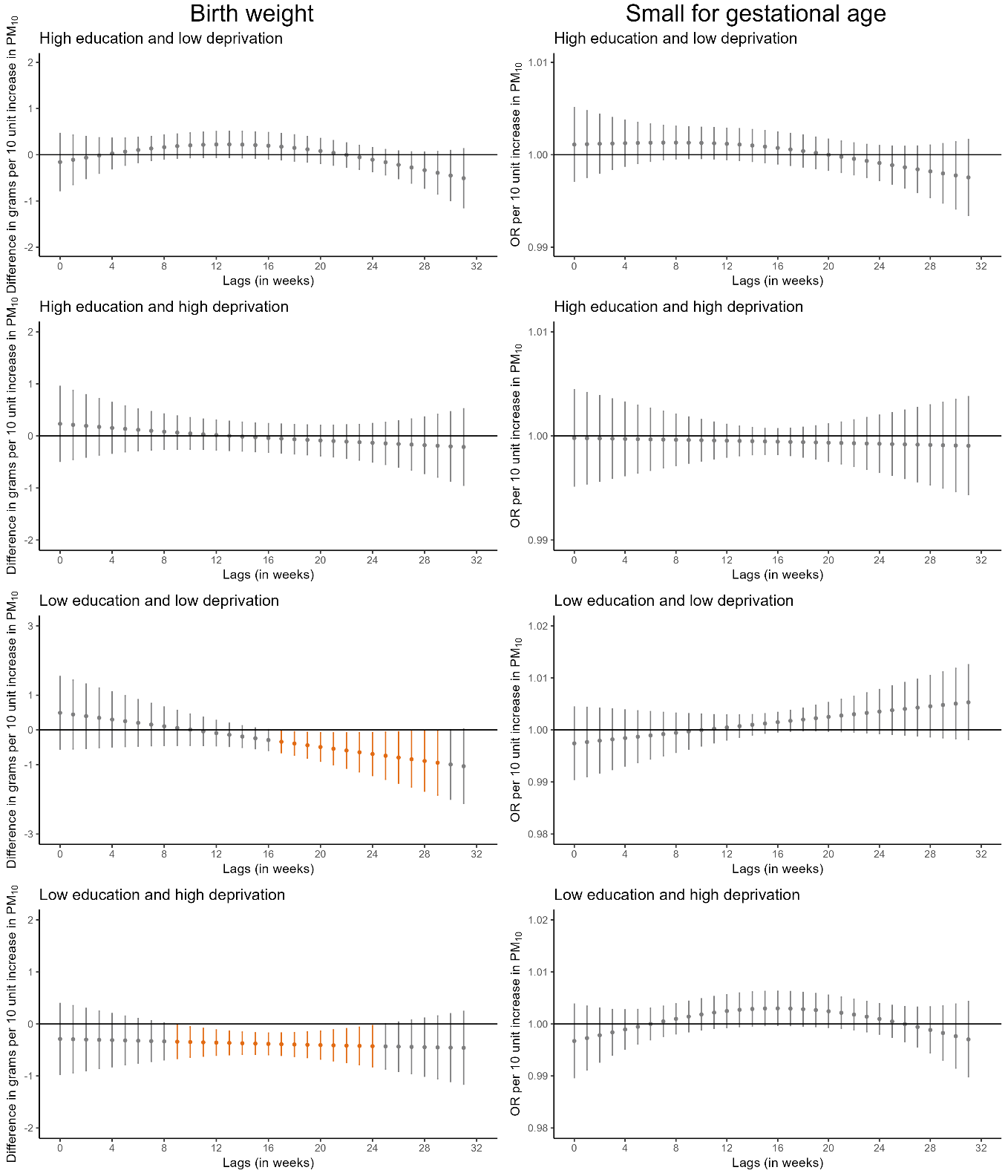


# **Figure S6. Adjusted lag-response association of weekly-average PM_10_ and PM_2.5_ concentrations during pregnancy with birthweight and small for gestational age according to the combination of maternal educational level and area-level deprivation index**

Abbreviations: CI, confidence interval; OR, odds ratio; PM_10_, particular matter with aerodynamic diameter less than 10μm; PM_2.5_, particular matter with aerodynamic diameter less than 2.5μm

Adjusted for parental age, paternal educational level, parental social class based on occupation, maternal nationality, maternal civil status, parity, month and year of conception, weekly temperature across pregnancy, and geographical region

Dots represent the effect estimates of the association between the exposure at each specific lag and the outcome. Vertical gray, blue, and orange lines represent 95% CI and indicate no divergence from the null, significant divergence from positive association, and significant divergence from negative association, respectively. All associations survived correction for multiple testing (p-value ≤ 0.05)


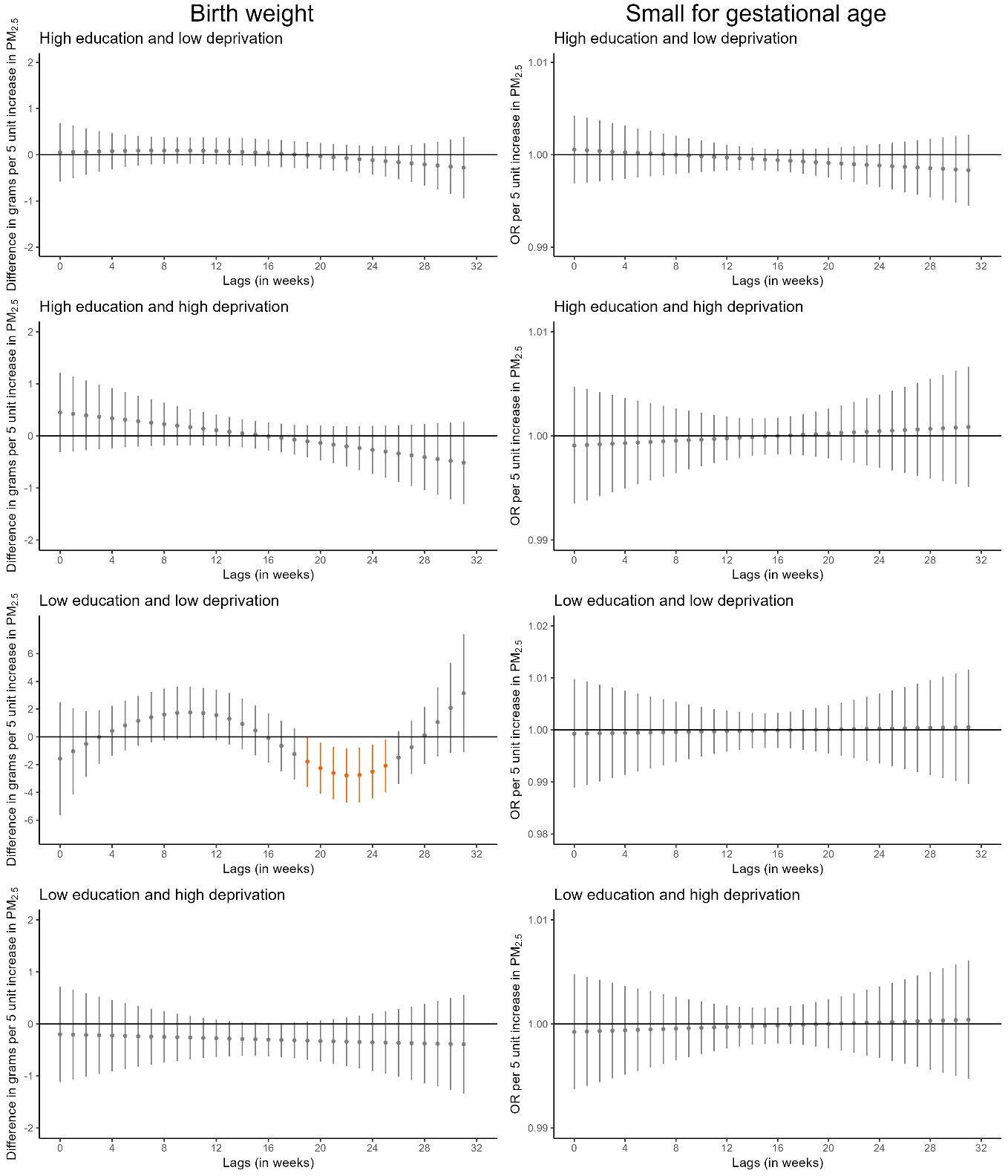


**Figure S6.** **Continuation**

# **Table S15. Adjusted lag-response association of weekly-average PM_10_ and PM_2.5_ concentrations with birthweight and small for gestational age according the combination of maternal educational level and area-level deprivation index**

|  | **Birthweight** | | | **Small for gestational age** | | |
| --- | --- | --- | --- | --- | --- | --- |
|  | **Lags** | **B** | **(95% CI)** | **Lags** | **OR** | **(95% CI)** |
| **PM_10_ (∆ 10 μg/m^3^)** |  |  | |  |  | |
| By maternal educational level & area-level deprivation index |  |  |  |  |  |  |
| High education / Low deprivation | --- | --- | --- | --- | --- | --- |
| High education / High deprivation | --- | --- | --- | --- | --- | --- |
| Low education / Low deprivation | Weeks 18-30 | -8.4 | (-15.7, -1.1) | --- | --- | --- |
| Low education / High deprivation | Weeks 10-25 | -6.2 | (-9.2, -3.1) | --- | --- | --- |
| **PM_2.5_ (∆ 5 μg/m^3^)** |  |  | |  |  | |
| By maternal educational level & area-level deprivation index |  |  |  |  |  |  |
| High education / Low deprivation | --- | --- | --- | --- | --- | --- |
| High education / High deprivation | --- | --- | --- | --- | --- | --- |
| Low education / Low deprivation | Weeks 20-26 | -16.8 | (-28.1, -5.4) | --- | --- | --- |
| Low education / High deprivation | --- | --- | --- | --- | --- | --- |

Abbreviation: B, Beta coefficient, CI, Confidence interval, OR, Odds ratio, PM_2.5_, particular matter with aerodynamic diameter less than 2.5μm

Adjusted for parental age, paternal educational level, parental social class based on occupation, maternal nationality, maternal civil status, parity, urbanicity, month and year of conception, mean temperature across pregnancy, and geographical region

# **Table S16. Adjusted association between pregnancy-average particle matter** **concentrations and birth outcomes, overall and according to level of urbanicity of the maternal address**

|  | **Birthweight** | | **Birthweight at term** | | **Low birthweight at term** | | **Preterm birth** | | **Small for gestational age** | |
| --- | --- | --- | --- | --- | --- | --- | --- | --- | --- | --- |
|  | **B** | **(95% CI)** | **B** | **(95% CI)** | **OR** | **(95% CI)** | **OR** | **(95% CI)** | **OR** | **(95% CI)** |
| **PM_10_ (∆ 10 μg/m^3^)** | (N=3,678,445) | | (N=3,468,035) | | (N=3,468,035) | | (N=3,678,445) | | (N=3,678,445) | |
| Overall association | **-7.1** | **( -8.5, -5.7)*** | **-4.9** | **( -6.1, -3.7)*** | 1.01 | (0.99, 1.03) | **1.04** | **(1.02, 1.05)*** | **1.01** | **(1.00, 1.02)** |
| By level of urbanicity |  |  |  |  |  |  |  |  |  |  |
| Cities | **-6.6** | **( -8.4, -4.8)*** | **-3.9** | **( -5.5, -2.2)*** | 1.01 | (0.99, 1.04) | **1.05** | **(1.03, 1.06)*** | **1.01** | **(1.00, 1.02)** |
| Towns or suburbs | **-6.8** | **( -9.3, -4.3)*** | **-4.4** | **( -6.7, -2.2)*** | 0.99 | (0.96, 1.02) | **1.04** | **(1.01, 1.06)*** | 1.00 | (0.98, 1.02) |
| Rural areas | **-7.7** | **(-12.5, -3.0)*** | **-7.9** | **(-12.2, -3.7)*** | 0.99 | (0.93, 1.04) | 1.01 | (0.97, 1.05) | 0.98 | (0.94, 1.01) |
| **PM_2.5_ (∆ 5 μg/m^3^)** | (N=1,991,031) | | (N=1,880,547) | | (N=1,880,547) | | (N=1,991,031) | | (N=1,991,031) | |
| Overall association | **-3.8** | **( -5.9, -1.7)*** | -1.4 | ( -3.3, 0.4) | 0.99 | (0.96, 1.01) | **1.04** | **(1.02, 1.06)*** | 1.01 | (0.99, 1.02) |
| By level of urbanicity |  |  |  |  |  |  |  |  |  |  |
| Cities | -1.8 | ( -4.7, 1.0) | 0.5 | ( -2.0, 3.1) | 1.00 | (0.96, 1.03) | **1.05** | **(1.02, 1.07)*** | 1.01 | (0.99, 1.03) |
| Towns or suburbs | **-6.8** | **( -10.6,-3.1)*** | -2.5 | ( -5.8, 0.8) | **0.95** | **(0.90, 0.99)** | **1.06** | **(1.03, 1.10)*** | 1.02 | (0.99, 1.04) |
| Rural areas | -3.5 | (-11.0, 4.1) | -3.8 | (-10.5, 3.0) | 1.00 | (0.91, 1.09) | 1.00 | (0.93, 1.07) | 0.96 | (0.91, 1.01) |

Abbreviation: B, Beta coefficient, CI, Confidence interval, PM_10_, particular matter with aerodynamic diameter less than 10μm; PM_2.5_, particular matter with aerodynamic diameter less than 2.5μm

Adjusted for parental age, parental educational level, parental social class based on occupation, maternal nationality, maternal civil status, parity, area-level deprivation index, urbanicity, month and year of conception, mean temperature across pregnancy, and geographical region. Stratified models were not adjusted for urbanicity.

In bold, associations with a p-value ≤ 0.05. *Associations that survive correction for multiple testing (p-value ≤ 0.017)

# **Table S17. Adjusted association of pregnancy-average PM_2.5_ concentrations with birthweight and preterm birth below and above 10 μg/m^3^, overall and according to level of urbanicity of the maternal address**

|  | **Birthweight** | | **Preterm birth** | |
| --- | --- | --- | --- | --- |
|  | **B** | **(95% CI)** | **OR** | **(95% CI)** |
| **PM_2.5_ ≤ 10 μg/m^3^ (∆ 5 μg/m^3^)** | (N=220,352) | | (N=220,352) | |
| Overall association | 1.9 | (-12.9, 16.7) | 0.90 | (0.79, 1.03) |
| By level of urbanicity |  |  |  |  |
| Cities | **35.7** | **( 3.6, 67.9)** | **0.64** | **(0.49, 0.81)*** |
| Towns or suburbs | 13.2 | (-16.4, 43.1) | 0.98 | (0.75, 1.28) |
| Rural areas | -16.5 | (-37.5, 4.5) | 0.99 | (0.82, 1.20) |
| **PM_2.5_ > 10 μg/m^3^ (∆ 5 μg/m^3^)** | (N=1,771,679) | | (N=1,771,679) | |
| Overall association | **-5.4** | **( -7.7, -3.1)*** | **1.07** | **(1.04, 1.09)*** |
| By level of urbanicity |  |  |  |  |
| Cities | **-3.7** | **( -6.6, -0.8)*** | **1.07** | **(1.04, 1.09)*** |
| Towns or suburbs | **-9.0** | **(-13.1, -4.9)*** | **1.08** | **(1.04, 1.12)*** |
| Rural areas | -2.6 | (-14.8, 9.5) | 1.06 | (0.95, 1.17) |

Abbreviation: B, Beta coefficient, CI, Confidence interval, OR, Odds ratio, PM_2.5_, particular matter with aerodynamic diameter less than 2.5μm

Adjusted for parental age, parental educational level, parental social class based on occupation, maternal nationality, maternal civil status, parity, area-level deprivation index, urbanicity, month and year of conception, mean temperature across pregnancy, and geographical region. Stratified models were not adjusted for urbanicity.

In bold, associations with a p-value ≤ 0.05. *Associations that survive correction for multiple testing (p-value ≤ 0.017)


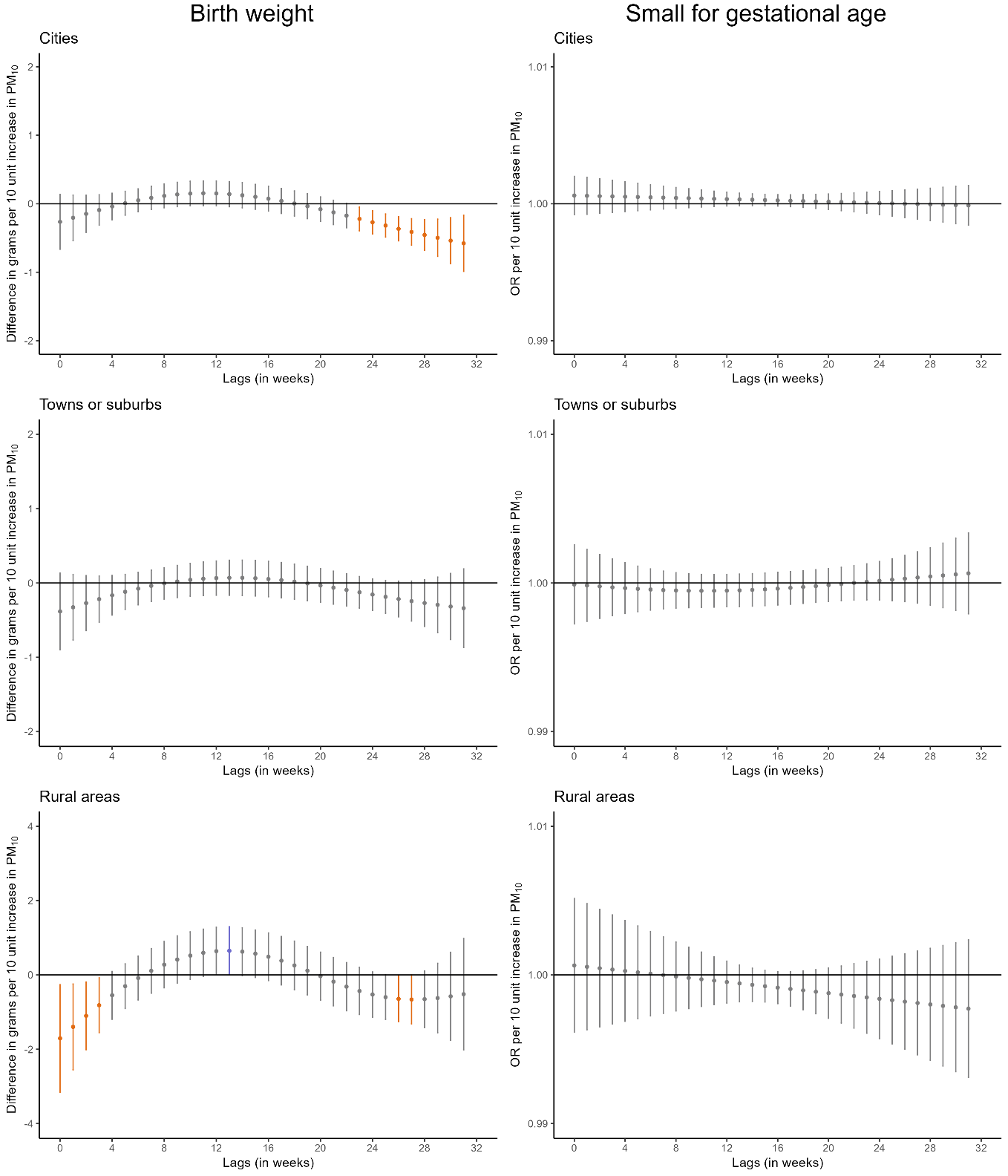


# **Figure S7. Adjusted lag-response association of weekly-average PM_10_ and PM_2.5_ concentrations during pregnancy with birthweight and small for gestational age according to level of urbanicity of the maternal address**

Abbreviations: CI, confidence interval; OR, odds ratio; PM_10_, particular matter with aerodynamic diameter less than 10μm; PM_2.5_, particular matter with aerodynamic diameter less than 2.5μm

Adjusted for parental age, parental educational level, parental social class based on occupation, maternal nationality, maternal civil status, parity, area-level deprivation index, month and year of conception, weekly temperature across pregnancy, and geographical region

Dots represent the effect estimates of the association between the exposure at each specific lag and the outcome. Vertical gray, blue, and orange lines represent 95% CI and indicate no divergence from the null, significant divergence from positive association, and significant divergence from negative association, respectively. All associations survived correction for multiple testing (p-value ≤ 0.05)


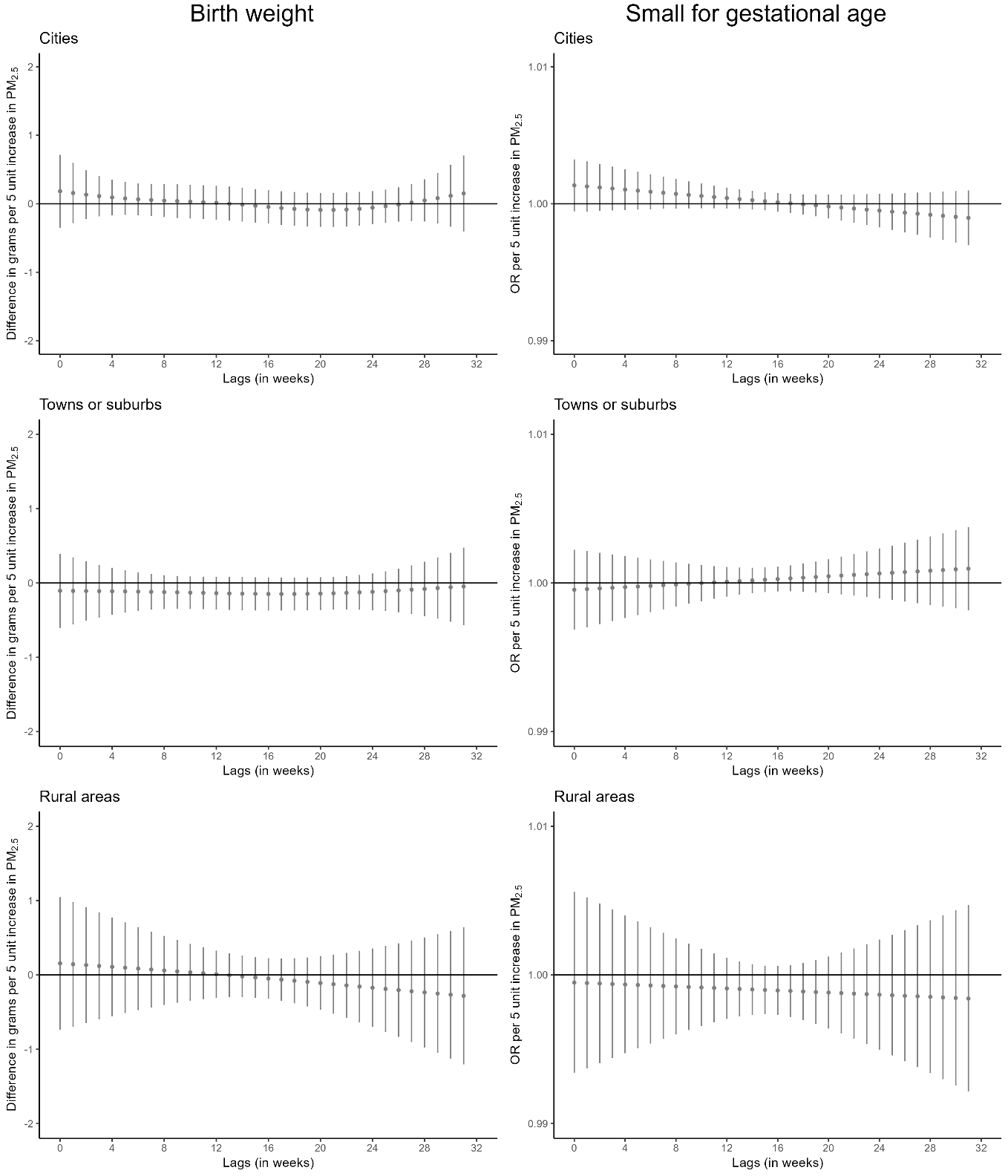


**Figure S7.** **Continuation**

# **Table S18. Adjusted association of pregnancy-average PM_10_ and PM_2.5_ concentrations with** **gestational-age-adjusted birthweight z-score**

|  | **Gestational-age-adjusted**  **birthweight z-score** | |
| --- | --- | --- |
|  | **B** | **(95% CI)** |
| **PM_10_ (∆ 10 μg/m^3^)** | (N=3,678,445) | |
| Overall association | **-0.004** | **(-0.007, -0.001)** |
| **PM_2.5_ (∆ 5 μg/m^3^)** | (N=1,991,031) | |
| Overall association | 0.003 | (-0.002, 0.007) |

Abbreviation: B, Beta coefficient, CI, Confidence interval, PM_2.5_, particular matter with aerodynamic diameter less than 2.5μm, PM_10_, particular matter with aerodynamic diameter less than 10μm

Adjusted for parental age, parental educational level, parental social class based on occupation, maternal nationality, maternal civil status, parity, area-level deprivation index, urbanicity, month and year of conception, mean temperature across pregnancy, and geographical region

In bold, associations with a p-value ≤ 0.05.

# **Table S19. Adjusted association between pregnancy-average particle matter concentrations and birth outcomes additionally adjusted for child sex**

|  | **Birthweight** | | **Birthweight at term** | | **Low birthweight at term** | | **Preterm birth** | | **Small for gestational age** | |
| --- | --- | --- | --- | --- | --- | --- | --- | --- | --- | --- |
|  | **B** | **(95% CI)** | **B** | **(95% CI)** | **OR** | **(95% CI)** | **OR** | **(95% CI)** | **OR** | **(95% CI)** |
| **PM_10_ (∆ 10 μg/m^3^)** | (N=3,678,445) | | (N=3,468,035) | | (N=3,468,035) | | (N=3,678,445) | | (N=3,678,445) | |
| Overall association | **-7.0** | **( -8.4, -5.6)*** | **-4.8** | **( -6.0, -3.6)*** | 1.01 | (0.99, 1.02) | **1.04** | **(1.02, 1.05)*** | **1.01** | **(1.00, 1.02)** |
| **PM_2.5_ (∆ 5 μg/m^3^)** |  |  |  |  |  |  |  |  |  |  |
| Overall association | **-3.6** | **( -5.7, -1.5)*** | -1.2 | ( -3.1, 0.7) | 0.98 | (0.96, 1.01) | **1.04** | **(1.03, 1.06)*** | 1.01 | (0.99, 1.02) |

Abbreviation: B, Beta coefficient, CI, Confidence interval, PM_10_, particular matter with aerodynamic diameter less than 10μm; PM_2.5_, particular matter with aerodynamic diameter less than 2.5μm

Adjusted for child sex, parental age, parental educational level, parental social class based on occupation, maternal nationality, maternal civil status, parity, area-level deprivation index, urbanicity, month and year of conception, mean temperature across pregnancy, and geographical region.

In bold, associations with a p-value ≤ 0.05. *Associations that survive correction for multiple testing (p-value ≤ 0.017)


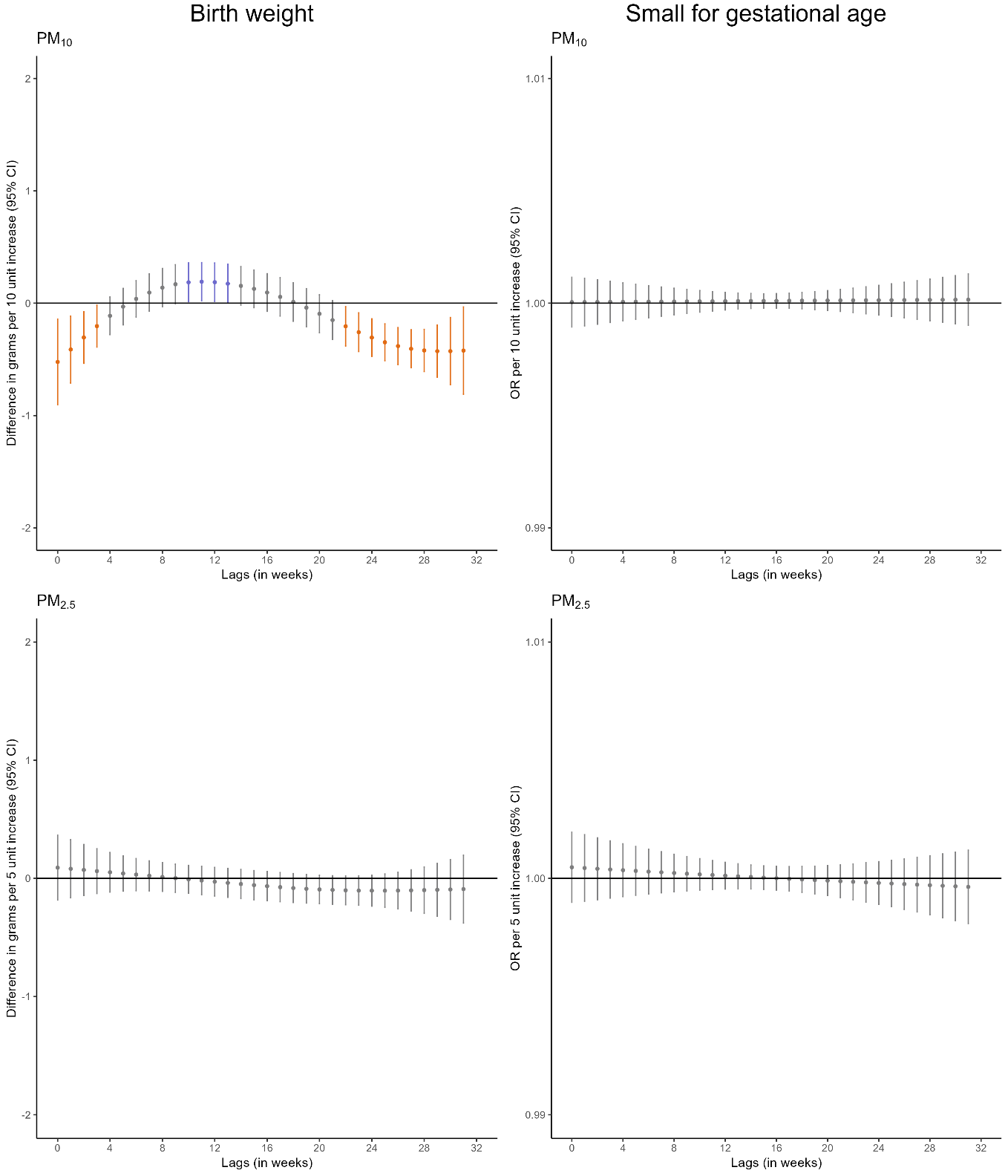


# **Figure S8. Adjusted lag-response association of weekly-average particle matter concentrations during pregnancy with birthweight and small for gestational age additionally adjusted for child sex**

Abbreviations: CI, confidence interval; OR, odds ratio; PM_10_, particular matter with aerodynamic diameter less than 10μm; PM_2.5_, particular matter with aerodynamic diameter less than 2.5μm

Adjusted for child sex, parental age, parental educational level, parental social class based on occupation, maternal nationality, maternal civil status, parity, area-level deprivation index, urbanicity, month and year of conception, weekly temperature across pregnancy, and geographical region

Dots represent the effect estimates of the association between the exposure at each specific lag and the outcome. Vertical gray, blue, and orange lines represent 95% CI and indicate no divergence from the null, significant divergence from positive association, and significant divergence from negative association, respectively. All associations survived correction for multiple testing (p-value ≤ 0.05)

# **References**

1. Lyapustin A, Wang Y, Korkin S, Huang D. MODIS Collection 6 MAIAC algorithm. Atmos Meas Tech. 2018;11:5741–65.

2. MACC-II Collaborative Group. Final report MACC-II: monitoring atmospheric composition and climate - interim implementation [Internet]. 2014 [cited 2024 Apr 26]. Available from: https://atmosphere.copernicus.eu/sites/default/files/repository/MACCII_FinalReport_0.pdf

3. Dee DP, Uppala SM, Simmons AJ, Berrisford P, Poli P, Kobayashi S, et al. The ERA-Interim reanalysis: configuration and performance of the data assimilation system. Quarterly Journal of the Royal Meteorological Society [Internet]. 2011 [cited 2024 Apr 26];137:553–97. Available from: https://onlinelibrary.wiley.com/doi/full/10.1002/qj.828

4. Pey J, Querol X, Alastuey A, Forastiere F, Stafoggia M. African dust outbreaks over the Mediterranean Basin during 2001-2011: PM10 concentrations, phenomenology and trends, and its relation with synoptic and mesoscale meteorology. Atmos Chem Phys. 2013;13:1395–410.

5. Elvidge CD, Baugh K, Zhizhin M, Hsu FC, Ghosh T. VIIRS night-time lights. Int J Remote Sens [Internet]. 2017 [cited 2024 Apr 26];38:5860–79. Available from: https://www.tandfonline.com/doi/abs/10.1080/01431161.2017.1342050

6. Kottek M, Grieser J, Beck C, Rudolf B, Rubel F. World Map of the Köppen-Geiger climate classification updated. Meteorologische Zeitschrift. 2006;15:259–63.
